# Laser Peening Process and Its Impact on Materials Properties in Comparison with Shot Peening and Ultrasonic Impact Peening

**DOI:** 10.3390/ma7127925

**Published:** 2014-12-10

**Authors:** Abdullahi K. Gujba, Mamoun Medraj

**Affiliations:** 1 Department of Mechanical and Industrial Engineering, Concordia University, 1455 De Maisonneuve Blvd. W., Montreal, QC H3G 1M8, Canada; E-Mail: a_gujba@encs.concordia.ca; 2 Department of Mechanical and Materials Engineering, Masdar Institute, Masdar City, P.O. Box 54224, Abu Dhabi, UAE

**Keywords:** laser shock peening, shot peening, ultrasonic impact peening, compressive residual stresses, hardness, microstructure, fatigue life

## Abstract

The laser shock peening (LSP) process using a Q-switched pulsed laser beam for surface modification has been reviewed. The development of the LSP technique and its numerous advantages over the conventional shot peening (SP) such as better surface finish, higher depths of residual stress and uniform distribution of intensity were discussed. Similar comparison with ultrasonic impact peening (UIP)/ultrasonic shot peening (USP) was incorporated, when possible. The generation of shock waves, processing parameters, and characterization of LSP treated specimens were described. Special attention was given to the influence of LSP process parameters on residual stress profiles, material properties and structures. Based on the studies so far, more fundamental understanding is still needed when selecting optimized LSP processing parameters and substrate conditions. A summary of the parametric studies of LSP on different materials has been presented. Furthermore, enhancements in the surface micro and nanohardness, elastic modulus, tensile yield strength and refinement of microstructure which translates to increased fatigue life, fretting fatigue life, stress corrosion cracking (SCC) and corrosion resistance were addressed. However, research gaps related to the inconsistencies in the literature were identified. Current status, developments and challenges of the LSP technique were discussed.

## 1. Introduction

Improvement of material surfaces has become an integral part of industrial operations; this is to improve the mechanical and metallurgical properties such as fatigue life, corrosion resistance, and wear and erosion resistance. However, among the recently advanced surface modification techniques is laser shock peening (LSP). LSP which dates back to late 60s and early 70s [1,2,3,4,5,6,7,8] has been described as a cold working process where pulses hit the surface through high power intensity and shock waves are generated. Experiments on materials using pulsed laser beam were first done at the Battelle Institute (Columbus, OH, USA) from around 1968 to 1981 [6,7]. The research work was carried out on aluminum and steel alloys for aerospace applications [7]. Subsequent experiments were carried out in laboratories in France such as CLFA (Cooperation Laser Franco-Allemande-Arcueil Cedex), LALP (Laboratoire d’Application des Lasers de Puissance-Arcueil Cedex) and LULI (Laboratoire d’Utilisation des Lasers Intenses-Ecole Polytechnique, Palaiseau Cedex) to further explore the industrial applicability of the process [6]. The process became known to the industry through two key patents. The first patent was registered in the United States by Industrial Materials Limited, 1973. The other patent was by Battelle Development Corporation, 1983. Later on, General Electric Company was credited with 23 US patents between 1996 and 2001 based on LSP [6]. Further historical perspective of LSP can be found in [7,9,10]. LSP is a versatile process able to modify different materials such as hard metals, soft metals and thin films. This technique is considered to be a potential substitute to the conventional shot peening (SP) process because of higher depth of residual stresses into the material surface reaching about 4–5 times deeper and higher intensity with uniformity across the surface. Furthermore, the LSP provides good surface finish as compared to SP where the roughness needs to be reduced by surface grinding or polishing for typical processes and applications that encounter wear [6,11,12,13]. Areas such as notches and fillets not accessible during shot peening can be treated by LSP [14,15]. More so, LSP can be compared with ultrasonic impact/shot peening (UIP/USP) [16,17,18,19,20,21,22,23,24,25,26] or ultrasonic impact treatment (UIT) [27,28,29,30,31,32,33,34,35,36,37,38,39,40] which has received considerable attention due to its ability to induce compressive residual stresses. This is achieved via electro-mechanical method where stress waves are generated in the materials by plastically deforming the surface [34,41]. For this process, handheld tools consisting of vibrating steel pins configured to frequency controller are employed to induce such stress waves [40,41]. UIP was initially developed in Russia in 1970s [35] and applied on welded joints in order to reduce welding residual stresses thereby enhancing the fatigue life [19,31,32]. Furthermore, the physics and mechanism of UIP/UIT has been addressed [19,34,35].

The applications of the LSP include improvement of fatigue life, stress corrosion cracking/corrosion resistance and wear resistance [42,43,44]. Similar to LSP, UIP has also been employed in enhancing fatigue life [16,25,27,29,30,33,39], fatigue strength [17,19,28,31,32], refinement of microstructure [20,22,23,24,36,38,39] and improvement of mechanical properties such as hardness [20,21,38]. Another application of LSP is the surface enhancement of thin membered sections as reported by Vaccari [9], Dane *et al.* [12] and Mannava *et al.* [45,46]. Potentially, the process can be integrated in the production line with high degree of automation [15]. LSP has also shown the suitability of treating complex geometries [47] due to laser beam delivery technology [48] whereas using UIP, this might be difficult to attain. This can be attributed to the handheld equipment usually used in UIP and the difficulty of having full contact with the surface [40,41].

It is worth mentioning that other review papers [6,7,9,49,50,51,52] tackling different aspects of the LSP process could be found in the literature. For instance, Fabbro *et al.* [51] and Peyre and Fabbro [7] reviewed the physics and applications of the laser shock process; whereas, Clauer [50] reported the fatigue resistance of material via laser shock peening, and Montross *et al.* [6] reviewed the LSP process with emphasis on its effect on microstructure and properties of metal alloys. This critical review discusses in more details the LSP process with emphasis on; shock wave generation, process parameters, characterization of laser peened specimens, current and future applications as well as the challenges for the LSP process. The effect of LSP process conditions on residual stress profiles, microstructure, surface roughness and mechanical properties are also considered. This paper also identified a wider spectrum of peened materials not previously mentioned in the aforementioned review articles. Furthermore, a comparative study between SP and LSP and UIP and LSP was performed, when possible. For instance, residual stress profile, surface roughness, stress relaxation, mechanical properties and applications were compared. The next section discusses how laser shock waves are generated, the process parameters, laser peened materials and characterization of laser peened samples.

## 2. Laser Shock Peening

LSP is a mechanical (cold working) process where pulses hit the surface with high power intensity and shock waves are generated [6,7,50,53]. These waves plastically deform the surface and compressive stresses are extended into the subsurface [7,53,54,55,56,57]. These dynamic compressive stresses are highest on the surface and decrease with depth into the material [58]. The next sub-section discusses how these shock waves are generated.

### 2.1. Generation of Laser Shock Waves

**Figure 1:**
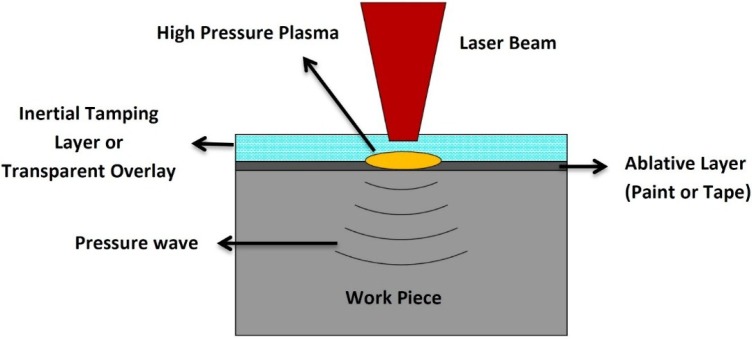
Schematic of Laser Shock Peening (LSP) Process.

The basic steps for laser shock wave generation can be described as the following sequence; (a) the target surface is covered with an absorbent (sacrificial) coating. This layer vaporizes, forming plasma on the surface with short duration pulse pressure. Absorbent layer prevents melting and laser ablation [3,6] while maintaining high surface quality [59] and without this layer, the energy conversion from pressure to shock cannot be made effectively [6]. This layer can be aluminum [53,60,61,62], copper [63], lead [4], vinyl tape [64,65,66], zinc [62] or black paint [6,11,54]. Hong *et al.* [64] showed experimentally that black paint layer has the best absorption ability to laser; where almost 100% laser energy is absorbed by black paint as compared to 80% absorption by Al. This means that when a target is irradiated, almost 100% of the laser intensity is used to generate the plasma; (b) transparent overlay (tamping layer) is applied to prevent the plasma from expanding away off the surface thereby increasing the intensity of the shock wave. These overlays, also known as the confining medium, can be water [11,52,53,54,65], quartz [4,5,64,67] or glass [64,68,69]. Other confining media are K9 glass [70], Pb glass [64], perspex [64] or silicon rubber [64]. The choice of confining medium solely depends on the substrate material, density and acoustic velocity which gives the so-called “acoustic impedance effect”. Finally, plastic deformation occurs when the shock wave stress exceeds the strength (dynamic yield strength, ${\text{σ}}_{y}^{dyn}$**)** of the metal [6,8,12,53,71]. This LSP process is schematically shown in Figure 1. The plastic deformation results in high dislocation multiplication and movements which affect the microstructure and properties of the material [6]. Peyre *et al.* [7] showed that increasing laser shock pressure increases the depth of plastic deformation. The description of how shock waves are generated is simple compared to the complexity of the process parameters and optimization. The next sub-section discusses the parameters involved in the LSP process and how the processing parameters can be a challenge to optimize.

### 2.2. Process Parameters for Laser Shock Peening

Effective shock peening process depends on the material (target), laser and beam parameters as well as the absorbent and transparent overlays properties. Typical requirements are: Q-switched laser system based on neodymium such as Nd-doped glass (Nd:Glass), yttrium aluminum garnet (Nd:YAG), yttrium orthovanadate (Nd:YVO_4_) or ytterbium-doped YAG (Yb:YAG) lasers. Different laser wavelengths of 1054 nm (infra-red), 532 nm (green), and 355 nm (ultraviolet) are used. Additionally, time duration of shock wave pressure (laser pulse length) ranges from 10 to 100 ns [6,52] with energy of 1–100 J per shot, and spot (diameter) size of 1–6 mm [6]. For the laser spot geometry; circular [4,11,65,71,72], elliptical [72,73], rectangular [73] and square [13,72] geometries have been studied with square showing better uniformity in intensity and good overlapping rate [72]. A frequency of less than 1 kHz and power density (*I*) in the range of 0.1–10^6^ GW/cm^2^ [6] are also required. Comparing with UIP, a higher frequency of 15–100 kHz [19] is employed. Furthermore, the most important LSP process parameters and equations have been summarized. Power density is expressed as a function of frequency, pulse time, power and spot area as given in Equation (1). Other parameters are the laser fluence as given in Equation (2), the load or pressure (P), which must exceed the dynamic yield strength for plastic deformation given by different authors in Equations (3)–(9). Equation (10) shows the reduced shock impedance (*Z*) for the target material (*Z*_1_) and confining medium (*Z*_2_) which is related to the density and speed of sound in the material. Table 1 lists typical values of acoustic shock impedance for various confining media and target materials. Furthermore, Equation (11) shows the Hugoniot Elastic Limit (*HEL*), which is the ultimate stress a material can withstand under compression without internal rearrangement [74]. The process optimization could be more complicated especially when many parameters are involved. For instance, shock pressure representation was inconsistent as found in the literature. Equations (3)–(9) indicate how the constants were varied based on the experimental observations. The variation of constants were attributed to the type of coating [7], confining medium [75] and target material properties [76,77]. This suggests that selecting the most effective processing parameters is paramount. The parameter selection can be achieved by extensive experimental work and simulations. Attempt has been made by *Society of Automotive Engineers* (SAE) to issue recommended parameters for laser peening of metals [47]. Most importantly, successful laser peening experiments and simulations have been conducted using different processing parameters as summarized in Table 2.

Intensity [54]:

(1) $$I\;(\frac{GW}{cm^{2}})=\;\frac{P_{avg}}{f\;\left(pt\right)\;a}$$

Laser Fluence [78]:

(2) $$Fluence\;\left(\frac{J}{cm^{2}}\right)=\;\frac{Laser\;pulse\;energy\;(J)\;}{focal\;spot\;area\;(cm^{2})}$$

Pressure [7,72,75,76,77,79]:

(3) $$P=A{\left(\frac{\text{α}}{2\text{α}+3}\right)}^{1/2}Z^{1/2}I^{1/2}$$

(4) $$P\left(GPa\right)=0.01{\left(\frac{\text{α}}{2\text{α}+3}\right)}^{1/2}Z^{1/2}I^{1/2}$$

(5) $$P\left(GPa\right)=1.02I^{1/2}$$

(6) $$P\left(Pa\right)=0.25{\left(IMA\right)}^{1/2}$$

(7) $$P=5.47{\left(\frac{\text{α}}{2\text{α}+3}\right)}^{1/2}I^{1/2}$$

(8) $$P\;\left(kbar\right)=BI^{1/2}$$

(9) $$P={\left(AZI\right)}^{1/2}$$

Reduced Impedance [77]:

(10) $$\frac{2}{Z}=\;\frac{1}{Z_{1}}+\;\frac{1}{Z_{2}}$$

Hugoniot Elastic Limit (*HEL*) [77]:

(11) $$HEL=\;\frac{1-\text{θ}}{1-2\text{θ}}{\text{σ}}_{y}^{dyn}$$

where, I is the laser power density in GW/cm^2^, $P_{avg}$ is the average power output in W, f is the laser frequency in Hz, pt is the pulse time in ns, *a* is the laser spot area in cm^2^, *B* is 21 or 10.1 for glass- or water-confined modes, respectively, *A* is absorption coefficient for surface coating, *M* is transmission coefficient for transparent overlay, α is the ratio of thermal to internal energy, Z is the reduced shock impedance of the target material and confinement medium, θ is Poisson’s ratio and ${\text{σ}}_{y}^{dyn}$ is dynamic yield strength at high strains.

**Table 1:** Typical values of *Z*_1_ and *Z*_2_ as reported by different authors.

Target material	*Z*_1_ × 10^6^ (g/cm^2^s) (Reference)	Confining medium	*Z*_2_ × 10^6^ (g/cm^2^s) (Reference)
Ti-6Al-4V	2.75 [65]	Water	0.17 [65]
AA7050-T7451	1.50 [77]	Perpex	0.32 [64]
SS304	3.61 [68]	Silicon rubber	0.47 [64]
Mg-Ca	0.88 [79]	K9 Glass	1.14 [64], 1.5 [68]
AISI 4140	3.96 [69]	Quartz Glass	1.31 [64]
Cu	0.16 [80]	Pb Glass	1.54 [64]
SS321	4.00 [81]	BK Glass	1.44 [69]

Typical processing parameters presented in Table 2 are power density, pulse duration, absorbent coating and transparent overlay. However, despite the fact that the same material is laser peened, the processing parameters varied for different authors. This indicates that the choice of processing parameters is solely confined to laser type, substrate condition and target application. The characterization of LSP treated surface will be instrumental in understanding the LSP process. The next section identifies some of the materials that have been laser peened.

### 2.3. Laser Peened Materials

Several materials have been peened successfully proving the effectiveness of LSP and the associated residual stresses induced. Typical research studies conducted are influence of LSP process on residual stress prolife, microstructure, mechanical properties, fatigue life, fretting fatigue life, corrosion and stress corrosion cracking behavior, as discussed in Section 3. Recent LSP studies have shown that laser peening technique can be used for shaping and forming of metals [47] and inspection of coatings [80] as discussed in Section 4. The materials laser peened include titanium alloys [11,13,53,54,55,65,66,71,72,82,83,84,85,86,87,88,89,90], aluminum alloys [3,5,60,61,62,64,75,76,90,91,92,93,94,95,96,97,98,99,100,101,102,103,104], different steel grades [8,66,69,70,79,97,105,106,107,108,109,110,111,112,113,114,115,116,117,118,119,120,121,122,123,124,125], copper alloys [126,127,128], zinc [67], nickel based alloys [116,129,130,131], super alloys [115,132,133], brass [91], magnesium alloys [80,134,135], bulk metallic glass [136] and other materials [4,63,137,138,139]. Comparatively, similar materials have been treated by the UIP technique such as soft steel [18], 304SS [33], Type 35 carbon steel [38], 316L SS [22], Inconel 718 [23], naturally exfoliated Al7075-T6511 [27], AlSi11Mg [31], Al2024-T351 [36], Al matrix composite reinforced with AlCuFe or Ti fine powders [20], Ti alloy [16], welded joints of steel grade 345W [28], steel grade A36 [29], 16Mn steel [17] and 304SS [19,37]. Despite treating similar materials only a few researchers [79,140] compared LSP and UIP processes. These comparisons will be addressed when possible in subsequent sections. Furthermore, Table 2 shows some of the process parameters and remarks made by different authors after laser peening on a variety of materials. The next section discusses the characterization of laser peened materials.

**Table 2:** Typical LSP processing parameters from the literature used for different materials with concluding remarks.

Material (Reference)	Power density (GW/cm^2^)	Pulse duration (ns)	Absorbent coating	Transparent overlay	Remarks
Ti-6Al-4V [53]	5	10	Al Foil	Water	The fatigue life was improved by 22.2% and 41.7% for one and two successive laser shocks respectively as compared to as-received sample.
Ti-17 [82]	3, 6, 9	9, 27	Al	Water	It was found that all LSP parameters had influence on the residual stress profile. LSP had no influence on roughness, little effect on work hardening and large effect on hardness due to the compressive stresses induced.
AA2195 [60]	5	18	Al	Water	The fatigue crack growth rate of stir welded joint was reduced using LSP as compared to SP. Furthermore, the reduced crack growth rate was comparable with unwelded material.
Al2024 T3 [83]	5	18	Black paint	Water	LSP specimen showed resistance to fatigue crack growth for various notch geometries as compared to unpeened specimen.
4140 Steel [84]	3	5	Al	BK7 Glass	WLSP and DSA showed improved fatigue life due to enhanced cyclic stability of compressive RS. The stability and reliability were attributed to the enhanced dislocation pinning effect associated with the WLSP and DSA process.
316 L SS [85]	2.5	10	No coating	Water	The potentiodynamic polarization investigation showed improvement in corrosion potential (*Ecorr*) and corrosion current density (*Icorr*) with increase in laser pulse density. LPwC specimen showed 30%–40% increase in microhardness than unpeened specimen.
2204 Duplex SS [86]	900, 1600, 2500 pul/cm^2^	8	No	Water	Greater RS were observed with higher pulse density. LSP showed improved crack growth rate resistance and fracture toughness as compared to untreated specimen.
Cu 15 µm foil [87]	6	10	Black paint	Water	The microscale laser dynamic forming (µLDF) showed that, materials are strengthened due to refined grains and large dislocation density which were dominant in the microstructure.
Alloy 22 [88]	10	25	Al	Water	Slitting method was used to establish a relationship between LSP parameters and RS on flat coupons. While contour method measured the spatial RS on critical geometrical features.
Inconel 600 [89]	10 J/cm^2^	12	-	Water Jacket	Tilted column microstructure was observed due to overlapping of laser spots during scanning. Spherical nanoparticles with 60 nm diameter were seen due to laser ablation.
Fe-3%Si [4]	0.1–1	25–200	Pb	Quartz	Laser shocking showed that quartz plus lead overlay gave the most deformation for a given power density. The deformation occurred by slip and twining mechanism. Most of the samples showed surface melting for 200 ns pulses.
Mg-Ca [79]	78	5 to 7	Black paint	Water	Sequential peening showed an increase in the tensile pile up region up to 50% which was considered applicable for orthopaedic applications.
AZ31B Mg [90]	5	23	Al7075	Water	Sub-grain sizes of 5.8 µm were obtained after four laser impacts. The RS near the surface increased with number of impacts. LSP showed retardation to SCC crack initiation and propagation.
Brass H62 [91]	1000, 2000, 3000 Pulses/cm^2^	10	-	Water	LSP showed that, the higher the pulse density the higher the microhardness, roughness and wear resistance. No observable features in the microstructure after LSP.
CMSX, AM1, Astroloy [92]	12–150, 90, 25	25, 33, 40	Black paint	Water, Glass	The deformation microstructure and macroscopic mechanical phenomenon studies were envisioned to provide optimum process parameters applied to fatigue and fretting fatigue specimens.

### 2.4. Characterization of Laser Shock Peened Materials

Due to the high strain rates of 10^6^·s^−1^ [76] involved in the laser shock peening process, it is usually accompanied with changes in microstructure and mechanical properties (hardening and strengthening). Despite the high strain rates during LSP, the total induced strain is much less than in the case of shot peening. Furthermore, these microstructural changes are characterized and investigated by means of TEM and SEM for the surface and sub-surface morphologies and features. For the TEM technique, typical information revealed are dislocation structure [11,79,91,141,142], dislocation entanglement [3,105,143], dislocation band [69] and precipitate structure [69,91,105]. SEM technique on the other hand, provides surface and subsurface features such as surface microcracks [82,83], macrocracks [144], fretting cracks [71], mild indentations [93], melting due to ablation [95] and other surface irregularities from the LSP process [54]. The measured compressive residual stress induced during the LSP is an important indicator of the effectiveness of the LSP process. Since residual stresses cannot be measured directly due to its extrinsic nature, strain in the crystal lattice which is an intrinsic property is usually measured. The residual stress are calculated from the strain while assuming a linear elastic distortion of the crystal lattice [145]. Several methods have been used to quantify the residual stress profile in the material. Among these methods are X-ray diffraction sin^2^ψ [85,145,146,147], hole drilling [85,92,93,148], slitting [86,129,130], neutron diffraction [129,130] and contour methods [60,61,94,106,130]. X-ray diffraction sin^2^ψ method is the most commonly used method to quantify the residual stresses. In this method, the strains are measured at particular diffraction angles and lattice planes where Bragg’s law is satisfied. Material constants such as Young’s modulus, Poison’s ratio as well as different tilt angles “ψ” are further employed to quantify the stress. For in-depth or subsurface residual stress profiles, measurements after material removal by electropolishing is used extensively [82,107]. The step-by-step material removal is in the order of microns and more details of this procedure have been reported in [145,146,147]. For the hole drilling method, a hole is drilled and variations of stresses are measured as a function of depth by a strain gage rosette [6,107]. Surface roughness measurement after LSP has been reported [8,76,86,108,149,150,151] as the arithmetic average (Ra) using surface profilometers such as Talysurf contacting surface [71] and electronic contact (stylus) [140]. The roughness can be areas of stress concentration and crack initiation sites which have adverse effect on fatigue life of components. Microhardness [54,62,82,95,108,109,110,134] and nanohardness [96,111,126,137] across the irradiated surface are also measured and reported in the literature. Increase in measured hardness has been attributed to plastic deformation resulting in dislocation multiplication and movement [49,76,85,128]. For in-depth or subsurface hardness measurements, nanohardness is a more reliable approach as compared to microhardness. This is because of the compressed layer thickness which might pose measurement difficulty when measuring microhardness. However, these characterization techniques give researchers an insight regarding the changes and modifications due to LSP processing. The general improvements can be further quantified after a series of mechanical testing depending on the application, such as test for fatigue resistance, fretting fatigue resistance, stress corrosion cracking, and water erosion resistance. The effect of LSP process on residual stress profile, microstructure, mechanical properties, stress corrosion cracking and corrosion behaviors will be discussed in the following section.

## 3. Effect of Laser Shock Peening

### 3.1. Residual Stress

**Figure 2:**
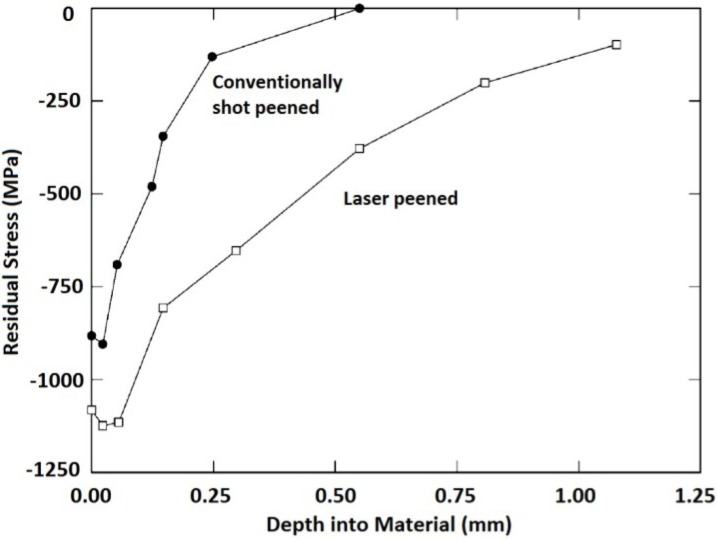
Residual stress profile in Inconel 718 induced by LSP and SP [99].

Induced compressive residual stress is the key output of the LSP technique as well as the conventional SP and UIP. The advantages of LSP over SP have been debated by a number of researchers and this debate arises from the beneficial effects of both peening processes. However, laser peening has shown more remarkable benefits related to in-depth and magnitude of compressive residual stresses. To epitomize the aforementioned LSP benefits over SP, Dane *et al.* [99] compared the induced residual stresses in Inconel 718 by both LSP and SP. They [99] showed that the LSP provided higher top surface and in-depth residual stresses as shown in Figure 2.

**Figure 3:**
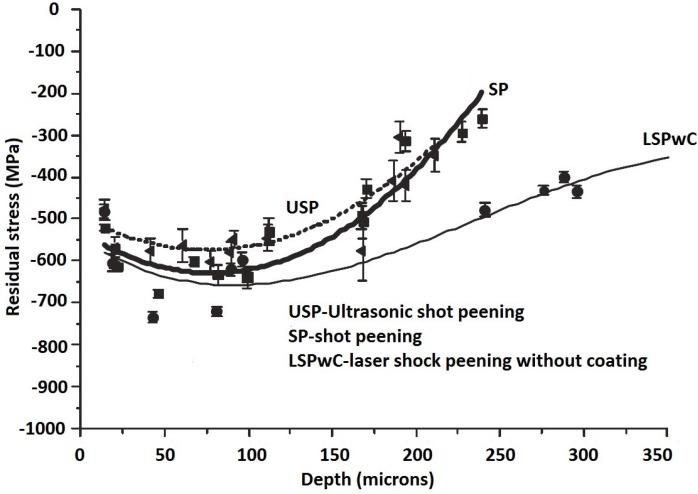
Residual stress-depth profiles in alpha phase of Ti-2.5Cu after LPwC, SP and ultrasonic shot peening (USP) [140].

The comparison between the effect UIP, SP and LSP without coating (LSPwC) on the residual stress profile could be found in the literature. It is worth mentioning here that LSP and LSPwC have the same working principles [85]. However, LSPwC employs the commercially available laser system with lower average power and pulse energy of 0.05–0.1 J [120,152] as compared to the conventional LSP with coating. For instance, Mordyuk *et al.* [81] compared the effects of UIP and LSPwC and confining medium on residual stress profile in the near-surface layer of AISI321. Their [81] compressive residual stress results showed that UIP and LSP induced residual stresses of −480 and −80 MPa, respectively. The lower RS from LSPwC could be due to the low level of intensity induced when no coating is applied. Again, the study of Maawad *et al.* [140] on UIP, SP and LSPwC of Ti-2.5Cu showed higher and deeper compressive residual stress after LSPwC compared to that after UIP and SP. The in-depth residual stress profile is shown in Figure 3. This observation is not in accord with the findings of Mordyuk *et al.* [81] which necessitates better understanding through more experiments. More so, a comparison between UIP, SP and LSP with coatings might reveal different residual stress profiles.

However, the stress profiles depend on surface condition, microstructure, material properties, laser peening parameters as well as the residual stress measurement techniques employed. It has been agreed upon that the compressive residual stresses are highest on the surface and decrease with depth into the material [58]. The choice of the residual stress measurement technique depends on sample geometry and desired stress profile. For instance, RS measurement on complex geometries can best be achieved using contour method [88]. Furthermore, Cellard *et al.* [82] study showed that all LSP parameters influenced the residual stresses induced in Ti-17 alloy. Nevertheless, it will be a cumbersome task to specify a single parameter that gives the optimal residual stress via LSP. For this reason, the optimization of LSP process is paramount for improved material performance. The following sub-sections describe the effect of individual LSP process parameters on the compressive residual stress profile.

#### 3.1.1. Residual Stress and Ablative Layer (Coating)

**Figure 4:**
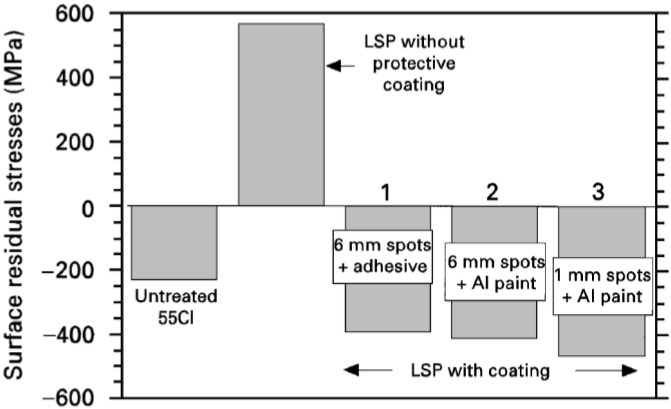
Average residual stress values determined at the surface of notched fatigue samples with different ablative layers and spot sizes peened with power density of 5 GW/cm^2^ [114].

As mentioned in the Section 2.1, the ablative layer (coating) has a direct effect on the induced residual stresses. Different authors have reported a variety of layers (coatings) on different materials as summarized in Table 2. Although, LSP is still possible without this layer [79,90,92,108,113,139,152], the induced compressive residual stresses in the material might not be effective or significant [6]. The reason for the ineffectiveness is that significant amount of the peening intensity is reduced without the ablative layer [81]. Another consequence of LSP without ablative layer is that tensile residual stresses are induced on the target surface due to melting [57,153]. For example, Peyre *et al.* [114] while demonstrating the effect of ablative coating with respect to the residual stresses on 55C1 steel in the water confining regime, stated that specimens coated had the highest compressive stresses whereas the absence of protective coating led to tensile stresses as shown in Figure 4. The tensile stresses could be attributed to the effect of laser ablation from the LSP process. Gonzalez *et al.* [57] studied the effect of absorbent overlay (black paint) on the residual stresses induced via LSP in 6061-T6 Al. They [57] concluded that the thermal effect was prevented on the metallic surface with coating for both perpendicular and parallel scanning directions. However, thermal effects were observed in the heat affected zone where no protective coating was employed thereby leading to tensile stresses [57]. This observation is in accord with what was reported by Peyre *et al* [114] as shown in Figure 4. Furthermore, since LSP is a mechanical process, it is paramount to eliminate the thermal effect and also select the best coating for particular laser peening process parameters and substrate conditions. This can only be realized through experimental and simulation attempts to further optimize the peening process and understand the effect of substrate surface conditions.

#### 3.1.2. Residual Stress and Confining Medium

The plasma formed as a result of sacrificial layer being vaporized is prevented from expanding away by the confining medium (transparent overlay). Thus, the confining medium increases the intensity of the shock wave generated which consequently leads to more plastic deformation. Table 2 shows a variety of confining media used by different authors. Water is the most widely used medium compared to glass and quartz. The reason for this could be due to its density and speed of sound (impedance) which is most compatible with most materials. Furthermore, water can also be suitable for peening complex geometries but might not be feasible when peening at higher temperature due to evaporation. Generally, the choice of confining medium depends on the density and speed of sound in the substrate (target) which is related to the reduced acoustic impedance given in Equation (10). However, study by Hong *et al.* [64] showed that the peak pressure generated is related to the confining medium and ablative layer but no reports on how the residual stress profiles are affected. Therefore, the lack of sufficient reports relating the effect of confining medium and induced residual stresses necessitates more fundamental understanding and additional future studies.

#### 3.1.3. Residual Stress and Laser Spot Size and Shape

The laser spot size and shape is another important parameter in the peening process. Circular shaped laser spots [11,86,91,111] are widely used while studies [13,72,114,130] have shown the possibility of using square shaped laser beam with improved features. Generally, the size of the laser spot can be varied which is confined to the selected power and power density [6]. This variation of size has an end effect on the shock wave propagation and consequently, the attenuation rate. Montross *et al.* [6] pointed out that for smaller spot diameter, the shock wave expansion was spherical and attenuation rate was 1/r^2^ while a planar front behavior and attenuation rate of 1/r were observed for larger spot diameter. Therefore, the less attenuation behavior in large spot diameter results in further propagation into the material. In addition, higher compressive stresses are expected for smaller spots due to the inverse proportionality of spot size and power intensity given in Equation (1). Trdan *et al.* [102] reported the compressive stresses generated in AA6082-T651 using 1.5, 2.0 and 2.5 mm spot diameter. Pulse density of 900 pulses/cm^2^ was adopted for all experiments. The results as shown in Table 3 suggest that 1.5 mm spot size generated higher compressive stresses compared to those of 2.0 and 2.5 mm. This was attributed to the mechanical effect of the waves resulting in high strain hardening with higher pulse densities. Similar residual stress results on aluminum alloys were reported by Sánchez-Santana *et al.* [151] and Kalainathan *et al.* [154].

**Table 3:** Near surface residual stress data (depth of 33 µm) [102].

Specimen	Spot size (mm)	δ_min_ (MPa)	δ_max_ (MPa)
1	-	22 ± 17	45 ± 22
2	1.5	−362 ± 31	−199 ± 27
3	2.0	−347 ± 28	−142 ± 23
4	2.5	−258 ± 24	−138 ± 22

Ganesh *et al.* [155] also suggested that larger spot sizes are preferred for deeper compressive zone. More so, larger spot sizes could show better coverage and uniformity due to overlapping of spots than smaller spot sizes. Based on the forgoing statements, the choice of either larger or smaller spot sizes depends on area coverage, uniformity and desired residual stress profile. Furthermore, understanding the effect of intensity profile on the residual stress distribution within a spot is paramount. This is because the intensity profile near the boundaries of the spot controls the release waves that are generated inside the specimen. This consequently affects the in-depth residual stress distribution, especially in thin parts.

#### 3.1.4. Residual Stress and Laser Power Density

**Figure 5:**
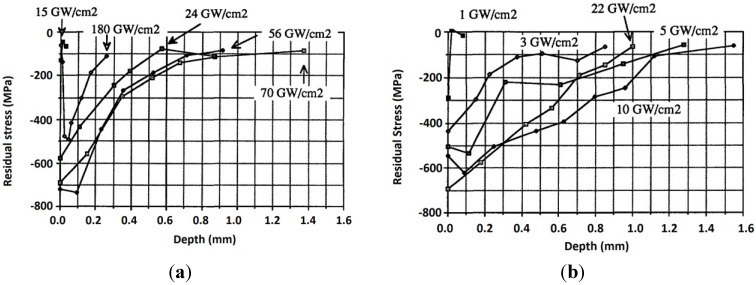
Stress distribution profile *vs.* depth after 2.5 ns (**a**) and 25 ns (**b**) laser irradiation [127].

The shock wave pressure, *P*, given in Equations (3)–(9) shows a direct proportionality with the power intensity. Thus, the surface residual stress increases with increasing pressure and the depth of these stresses increases when the power intensity threshold is exceeded [6,156]. Thus, it can be said that the magnitude and depth of compressive RS depends on energy density [135]. It is also worth mentioning that there is a critical power density beyond which defects such as microcracks might be introduced. For example, Liu *et al.* [113] observed internal cracking in 7050 aluminum alloy and attributed it to laser power density. They [113] concluded that high power density is a major factor in forming internal cracks in laser peened material. Furthermore, internal cracking within the compressed layer has been a challenge in peened materials and this will be discussed in Section 5 of this paper. Fournier *et al.* [127] reported the residual stress profiles for a pulse time of 2.5 and 25 ns using different power densities on 35CD4 and XC38 steel. The residual stress distribution *versus* depth as measured using X-ray sin^2^Ψ method is shown in Figure 5a,b. They [127] stated that the mechanical effect due to shock wave depends on surface load and pressure time profile. As observed in Figure 5b, more depth was reached with longer pulse time which corresponds to lower power density [157] and the compressive zone was increased compared to shorter pulse time with higher power density shown in Figure 5a. LSP process with 10 GW/cm^2^ and 25 ns showed the largest residual stress depth and compressed layer [127].

Furthermore, one can see that increasing power density increased the top surface compressive residual stress. For instance, in Figure 5b, top surface compressive residual stress of −500 MPa, −550 MPa and −700 MPa were observed for 22, 10 and 5 GW/cm^2^ experiments respectively. Similar residual stress trends were reported by Peyre *et al.* [76] in aluminum alloys (7075, Al12 and A356), Rubio-González *et al.* [86] in 2204 duplex SS, Zhang *et al.* [109] in 2024-T62 and Cao *et al.* [66] in 4140 steel, 12Cr, 316 L stainless steel and Ti-6Al-4V. The variation of stress at the top surface could be different when process conditions such as multiple shocks are introduced.

#### 3.1.5. Residual Stress and Multiple Laser Shocks and Overlaps

**Figure 6:**
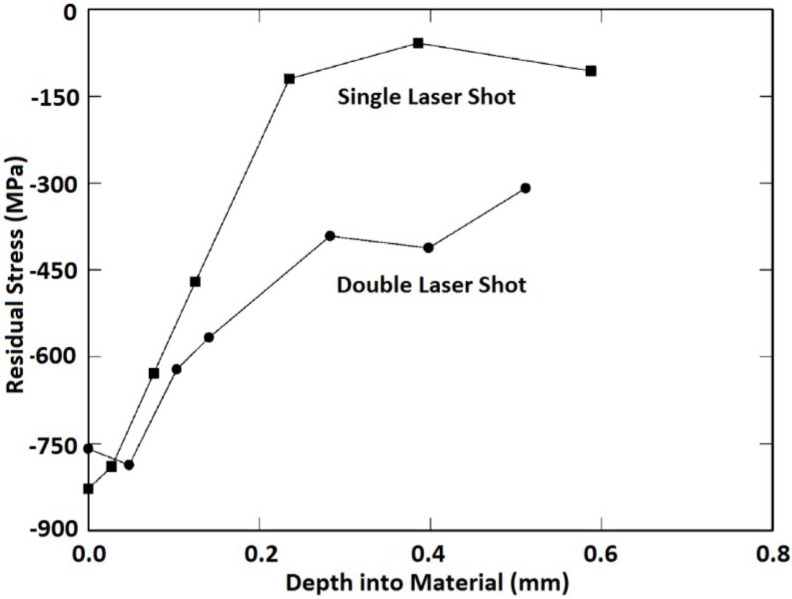
The depth of compressive stresses induced by single and double successive laser shocks on Ti-6Al-4V [99].

**Figure 7:**
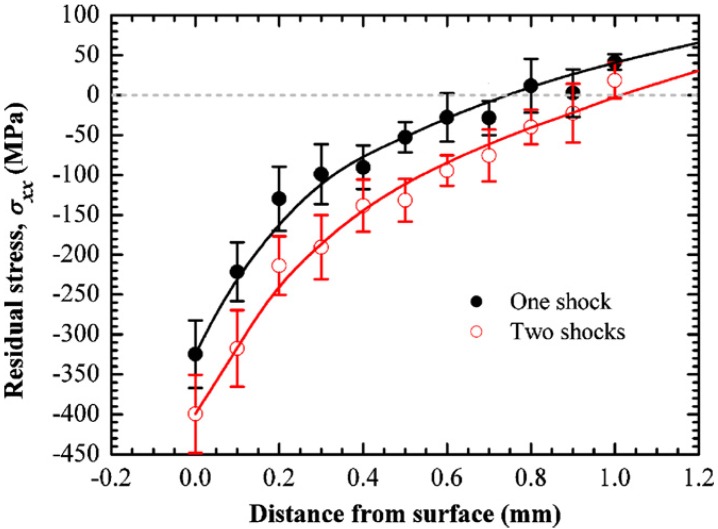
Residual stress profiles induced by one and two shocks using 40% overlapped spots on Ti-6Al-4V [53].

Multiple laser shocks and overlapping of spots to cover large areas [6] have shown significant influence on the residual stress profile. This is due to the fact that the higher the number of shocks the larger the induced plastic deformation until a saturation point is reached [158]. Furthermore, overlapped regions show a relatively uniform distribution of compressive residual stresses after LSP [11,76,107,159]. Dane *et al.* [99] compared the compressive residual stresses induced by single and double successive laser shocks on Ti-6Al-4V using laser fluence of 200 J/cm^2^ and a pulse duration of 30 ns. Results from their [99] work, using higher laser shocks showed deeper residual stresses than in the case of single laser shock as shown in Figure 6. Zhang *et al.* [53] investigated the effect of one and two laser shocks on Ti-6Al-4V with a 40% overlapping. The residual stresses were measured using X-ray diffraction sin^2^ψ method as shown in the Figure 7 and similar trend was observed earlier by Dane *et al.* [99]. The reason for deeper stresses in Dane *et al.* [99] and Zhang *et al.* [53] can be attributed to the plastic deformation that created more dislocation movements. Despite having two laser shocks in Figure 6 and Figure 7, compressive stresses of −750 MPa and −420 MPa were observed on the surface, respectively, and in-depth stresses reached 0.6 mm for [99] and 0.9 mm for [53]. The reason for the variations in residual stress profile could be due to process parameters, and the use of different overlap of 40% in [53] *versus* complete overlap in [86]. Another possibility is the measurement procedure used to quantify the residual stress induced at different lattice planes. From Figure 6, the residual stresses at the surface were lower in the double laser shocks, whereas, it was higher for single laser shock. This can be explained as the stress saturation point (−800 MPa) was reached using single laser shock or the stresses have been relaxed due to increase in number of shocks [66]. Furthermore, the absorbent layer may have been destroyed during multiple shocks. This variation in stresses shows that clear understanding and estimation of residual stresses and their dependence on the number of shocks [88] and percentage overlap is important and still requires further investigation.

#### 3.1.6. Residual Stress and Specimen Thickness

**Figure 8:**
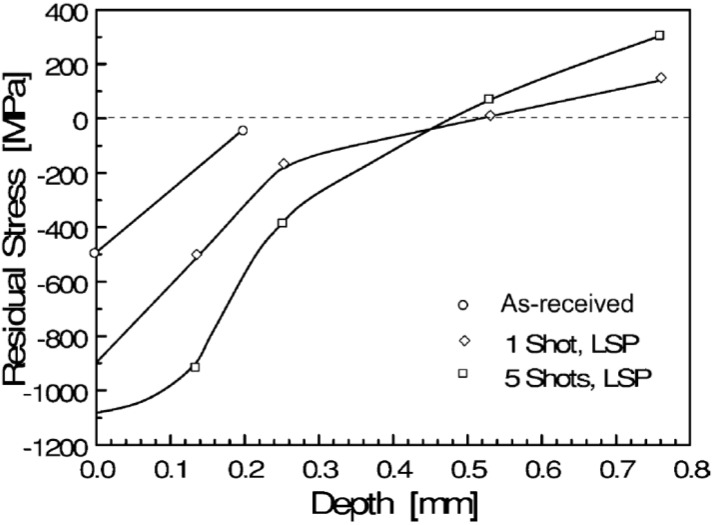
Residual stress profiles in 1.5 mm 4340 steel sheet for as received, one and five laser shocks [161].

It is practically feasible to laser peen both thin and thick specimens. However, Hammersley *et al.* [160] recommended that compressive residual stresses on any material top surface should be confined to 10% of the specimen thickness in order to avoid distortion. For thin sections, such as gas turbine compressor blades and plate valves, careful selection of processing parameters is required to concentrate the residual stresses at the top-most surface [160]. In this case, in depth compressive residual stress might not be increased even with increased peening intensity. For instance, Masse and Barreau [161] reported that increase in number of shocks from 1 to 5 showed no increase in the depth of compressive stresses in 1.5 mm thick 4340 steel but significantly increased the magnitude of the in-depth stresses as shown in Figure 8.

**Figure 9:**
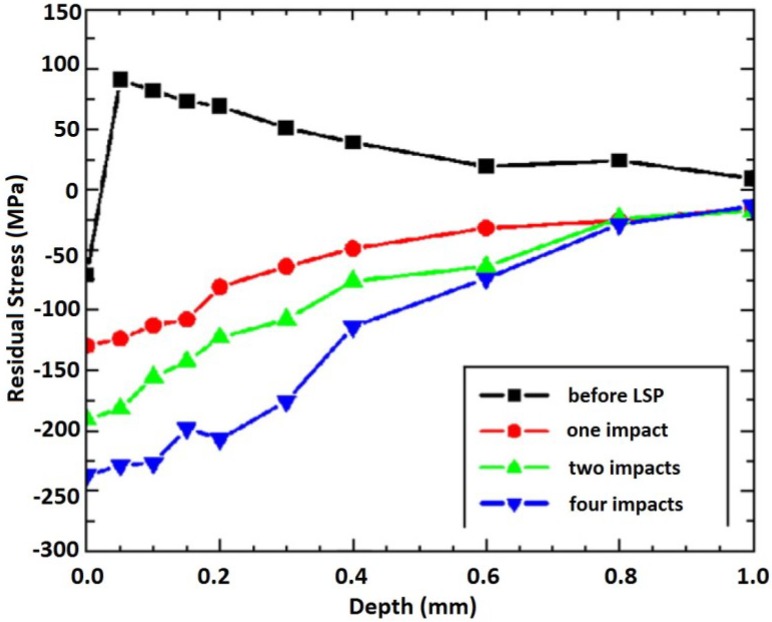
Residual stress (σ_x_) variation with depth in AZ31B Mg alloy after different number of laser impacts [90].

From Figure 8, it can be seen that for both peening conditions, the depth of compressive stresses was 0.5 mm and tensile stresses were more apparent half way through the specimen thickness after five shocks. These reflected tensile stresses after five shocks could result in the specimen distortion though this point has not been pointed out in the work of Masse and Barreau [161]. In laser peening of thin sections, meticulous attention should be given to the effect of tensile stresses which could eventually distort the part; this point also has been seen as a challenge in thin peened sections as indicated in Section 5. Therefore, peening of both sides could eliminate the distortion caused by tensile stresses since both reflected stresses will cancel out [65]. The peening can be achieved by either simultaneously peening both sides or carefully placing one side of the specimen on a supporting plate while peening the other. It is worth mentioning here that for irregular geometries, peening of both sides might not be suitable. The unsuitability can be attributed to the challenges related to set-up and residual stress profile measurement. For thick sections, increasing the number of impacts increases the compressed zone without consequently distorting the specimen. For instance, Zhang *et al.* [90] showed this observation on a 10 mm thick AZ31B Mg alloy using one, two and four laser impacts as shown in Figure 9.

From Figure 9, it can be seen that the compressive stress zone increased with increasing the number of impacts. This was attributed to the increased plastic deformation with higher number of impacts. Furthermore, for all studied conditions [90], the compressive stresses at 0.8 mm depth were approaching zero; this could be due to the stress attenuation effect. Therefore, based on Figure 8 and Figure 9, one can emphasize that increasing the number of impacts or shocks in either thin or thick specimens increases the magnitude of compressive residual stress. However, an increase in the depth of the compressive RS layer might not be achieved as shown in both Figure 8 and Figure 9. This can be due to reaching the saturation point. The saturation point is the plastic deformation limit point where the magnitude of the shock pressure becomes greater than twice the Hugoniot Elastic Limit (*HEL*) of the target material [76]. Again, the compressed layer was confined to 30% and 8% of the 1.5 mm and 10 mm thickness, respectively. Therefore, distortion could have been encountered especially for the 1.5 mm specimen. The maximum depth of the compressive residual stresses depends on the peening process parameters [82], surface condition of the material [5] as well as the specimen thickness and/or geometry [88].

#### 3.1.7. Residual Stress and Wavelength

**Figure 10:**
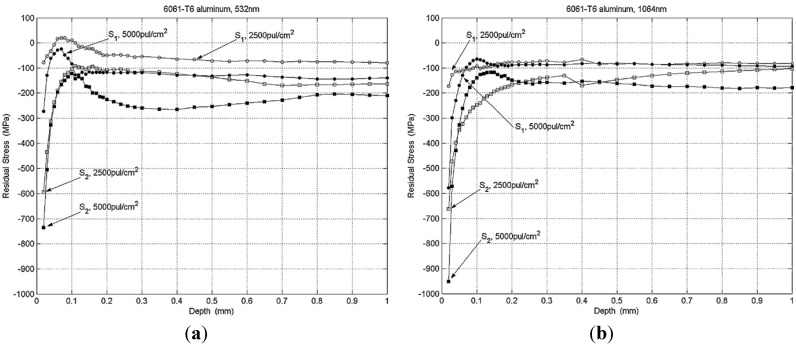
In-depth residual stress profile processed using (**a**) 532 nm and (**b**) 1064 nm wavelength [111].

For most of the LSP studies, 355 nm [63,67,82,100,126,127,162], 532 nm [66,87,97,98,101,114] and 1064 nm [53,54,62,66,72,92,95] laser wavelengths have been used. The effect of wavelength on residual stress profile has not been meticulously studied; this could be due to its dependency on the laser type, power density [51,163] and the beam characteristics. However, a few studies have been performed, for instance, Gomez *et al.* [111] studied the effect of LSP on 6061 Al using (1.2 J/pulse) 1064 nm and (0.9 J/pulse) 532 nm wavelengths in a 8 ns laser with a frequency of 10 Hz. The set-up was aided with flat mirror and a convergent lens to deliver the pulse from the laser. Pulse densities of 2500 and 5000 pules/cm^2^ were used for both wavelengths. The results in Figure 10a,b showed that using higher pulse energy and wavelength resulted in measuring as high residual stresses as −950 and −750 MPa for 1064 and 532 nm wavelengths, respectively. The residual stresses in both figures are confined to the top surface of the specimen [151]; this could be attributed to the short pulse duration of 8 ns used and another possibility is error from the hole drilling measurement method. X-ray method could give better in-depth stress measurement with electropolishing. Therefore, the LSP process configuration such as mirror and convergent lens positioning used to deliver pulse is vital and the effect of wavelength is an area worth further understanding through more experimental investigations.

#### 3.1.8. Residual Stress and Pulse Duration

**Figure 11:**
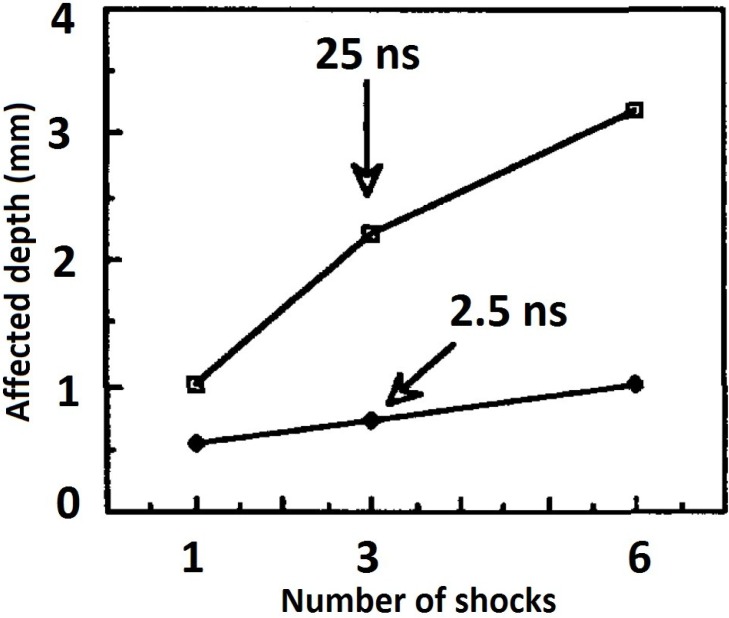
Residual stress depth as a function of pulse duration [127].

Pulse duration, usually in the nanosecond (ns) range, has been related to the shock wave pressure generated and consequently, the residual stress profile [59]. Montross *et al.* [6] mentioned that short pulse time results in insufficient shock pressure whereas too long time results in melting and damage of the substrate. More so, Hackel *et al.* [59] reported that for a power density of 5–10 GW/cm^2^ with short duration, ablation occurs rapidly and high pressure plasma is formed. Furthermore, the effect of pulse duration on the residual stress profile has been studied. For instance, Fournier *et al.* [127] reported the effect of using 2.5 ns and 25 ns pulse times on two steel grades. They [127] suggested that the affected depth was significantly dependent on the pulse duration especially when number of impacts were increased as shown in Figure 11.

From Figure 11, 25 ns experiment showed higher depth than 2.5 ns. However, other processing parameters such as power densities, ablative layer, and substrate condition could have played a more significant role. Although, an intermediate pulse duration between 2.5 and 25 ns using one or more shots could have revealed a better understanding of the relationship between pulse time and residual stress depth. Again, as presented in Table 2, it is evident that the choice of pulse duration must be made meticulously for optimized conditions, for instance, different pulse times were used for Ti-6Al-4V the same material [11,13,53,54,55,65,66,71,72,82,83,84,85,86,87,88,89,90].

#### 3.1.9. Residual Stress and Geometry

Many of the LSP research studies were carried out on simple geometries e.g., flat surfaces. Despite the feasibility of laser peening of complex geometries, more fundamental experimental studies are still desired. For example, Yang *et al.* [77] proposed a non-linear FEA approach for studying complex geometries. They [77] investigated the effect of compressive residual stresses on curved AA7050 rods. The diameters of the central sections were 4, 6, 8, 10 and 12 mm. Variations and discrepancies in radial and longitudinal residual stresses were observed which was attributed to the geometrical constraints. In some cases, the variations in residual stress profile might consequently lead to the variations in desired properties. The variation and location of residual stresses in complex geometrical components are still challenges yet to be addressed as mentioned in Section 5.

**Figure 12:**
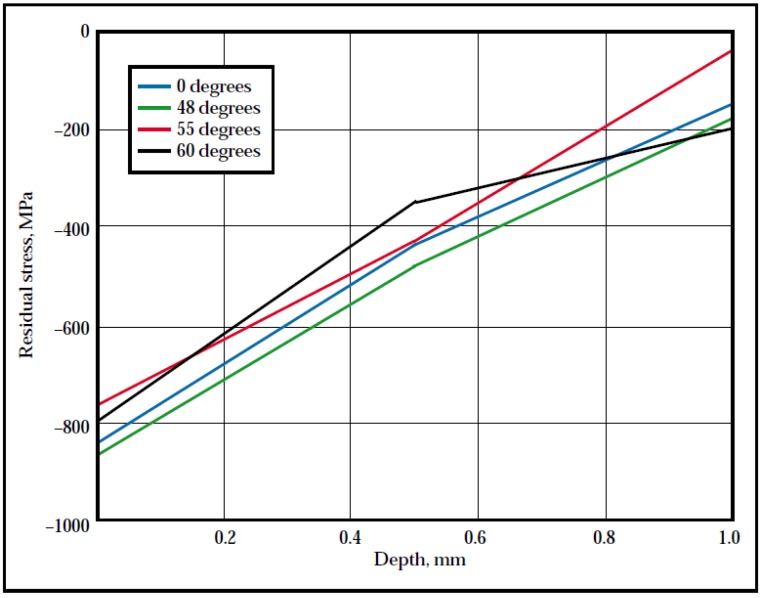
Residual stress profiles in laser peened Ti-6Al-4V for various laser incidence angles [164].

#### 3.1.10. Residual Stress and Laser Incidence Angle

The effect of laser beam incident angle on the compressive RS by LSP is rarely investigated and reported. Nevertheless, Hill *et al.* [164] in collaboration with Metal Improvement Company studied the effect of the incident angle on residual stress profile. Ti-6Al-4V (8.73 mm thick) was laser peened with incident beam angles varying from normal to the surface (0°) to 60°. The X-ray diffraction measurements in Figure 12 showed that the near surface residual stresses were dependent on the incident angle. However, no clear correlation was shown between the angles and observed in-depth residual stresses. Based on the forgoing result, peening can be done at high laser beam angles which can provide feasibility of peening complex geometries. However, more fundamental understanding will be paramount to substantially predict the stress profiles of such geometries as indicated in Section 3.1.9.

### 3.2. Surface Roughness

Surface roughness from all peening processes has shown significant effects during mechanical testing such as fatigue and fretting fatigue life testing [53,109]. For example, high surface roughness could provide more crack initiation sites which has adverse effects on fatigue life, corrosion [85] and wear resistance [165,166]. Besides, the LSP and SP process parameters and target surface conditions have been seen to influence the resulting surface roughness. For instance, Peyre *et al.* [76] compared the effect of peak pressure from LSP and SP on aluminum alloy in relation to the resulting surface roughness. Using a peak pressure of 0–6 GPa and 3–10 GPa for LSP and SP, respectively, they [76] recorded a higher surface roughness for shot peened than the laser peened specimens. One can argue here that irrespective of the surface treatments employed, the surface roughness is increased as compared to the baseline material. However, the final surface roughness depends on the initial starting roughness. Table 4 compares the surface roughness arithmetic mean (Ra) values for baseline, laser and shot peened specimens as reported by various authors [8,76,86]. From this table, higher roughness was observed after shot peening compared to laser peening and baseline material. This is due to the extreme and multiaxial loadings nature in shot peening than the uniaxial loading in laser peening [6]. Furthermore, due to the deeper layer of compressive stress zone after LSP, the surface roughness can be reduced to compare with the baseline material without significantly relieving the compressive stresses. This suggests that surface irregularities can be removed while retaining significant amount of the compressive RS. Contrarily, reduction of surface roughness after SP results in significant removal of compressive stress layer and relaxation.

**Table 4:** Arithmetic mean (Ra) values from different authors.

Material	Ra (µm) arithmetic mean values			
	Baseline	LSP	SP	Reference
A356	0.70	1.10	5.80	Peyre *et al.* [76]
AA7075	0.60	1.30	5.70	Peyre *et al.* [76]
316L SS	0.05	1.15	1.40	Peyre *et al.* [8]
Ti-6Al-4V	0.04	0.20	1.40	Liu and Hill [96]

In terms of application, LSP and SP have shown distinct advantages. For instance, in wear applications where surface roughness is important, LSP is a favorable process since significant amount of the compressed layer is retained after careful polishing while for SP, the high surface roughness renders the process for wear application not very beneficial [6]. In coating applications, SP process shows better adhesion of the coating to the substrate than LSP. This can be attributed to the higher roughness produced during SP process. In enhancing thin sections, the SP technique is not feasible because of the possibility of surface damage and irregularities [6]. In metal forming of components, deeper stresses from laser peening can create radius of curvatures 3 to 8 times greater and a surface 6 times smoother than SP [59]. Still, one can argue that both processes have their merits and demerits. This could be some of the reasons why researchers are not keen on adapting LSP to fully substitute the SP process. More on the present and future applications of LSP will be discussed in Section 4.

From the work of Maawad *et al.* [140] on UIP and LSPwC of Ti-2.5Cu. A higher surface roughness (Rz = 17 µm) was observed after LSPwC as compared to the surface roughness (Rz = 2.5 µm) after UIP. This observation could be due to the unprotected surface during LSPwC or the formation of surface defects such as microcracks. Also, this may suggest that the fatigue and fretting fatigue performance would be better for the case of UIP than LSPwC. However, due to the handheld apparatus used in UIP, one would also expect a higher surface roughness when compared to the conventional LSP. Though, this observation has not been reported so far.

### 3.3. Microstructure

**Figure 13:**
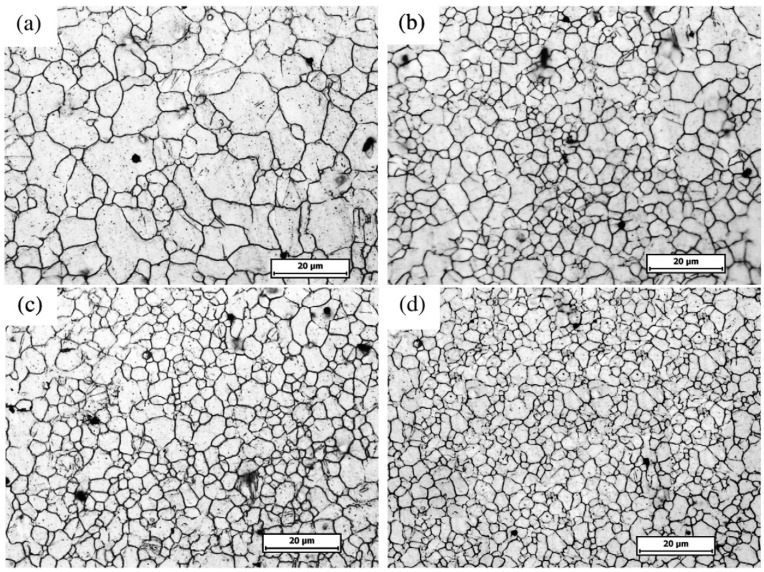
Optical macrographs of AZ31B Mg alloy surface (**a**) before LSP; (**b**) one impact; (**c**) two impacts; and (**d**) four impacts [90].

LSP treated material undergoes high strain rates of 10^6^·s^−1^ [148] due to plastic deformation thereby resulting in microstructural changes near the metal surface [53]. This results in refinement of the grains [43] and have been linked to the enhanced material properties such as hardness [107] which translates to higher fatigue strength [11], tensile strength [3] and wear resistance [43]. The refinement of microstructure is also a phenomenon observed in UIP treated materials [20,22,23,24,36,38,39] thereby improving the material properties. Furthermore, Zhang *et al.* [53] stated that the microstructural features are dependent on the LSP processing parameters. Zhang *et al.* [90] reported the effect of one, two and four laser impacts on the microstructure of AZ31B Mg alloy. From their [90] results shown in Figure 13, it is evident that the increase in number of impacts results in more plastic deformation thereby refining the grains. This type of refined microstructure has shown significant improvement in crack arrest and prevention of stress corrosion cracking as will be discussed in Section 3.5.2. Therefore, depending on the LSP conditions, target material and heat treatment of the alloy, varying microstructural features can be observed which could either be detrimental or beneficial. For instance, no detrimental effects could be observed by SEM in the works of [11,110] while defects such as microcracks [93], craters, holes and solidified droplets were observed by others [95]. Furthermore, other features observed include mild indentations [93], formation of ɛ-hexagonal close-packed (hcp) martensite in Hadfield steel [129], tilted column due to overlapping in Inconel 600 [89], deformation mechanism by slip and twinning [4], internal cracking [113], and tangled dislocation structure [63,69,79,105,128]. From the aforementioned microstructural features and characteristics, one can say that there is still lack of fundamental understanding at the metallurgical and microstructural levels regarding LSP treated materials. Careful selection of process and material conditions as well as the in-situ monitoring of the process are recommended to obtain better control over the resulting microstructure.

### 3.4. Mechanical Properties

#### 3.4.1. Hardness, Elastic Modulus and Yield Strength

**Figure 14:**
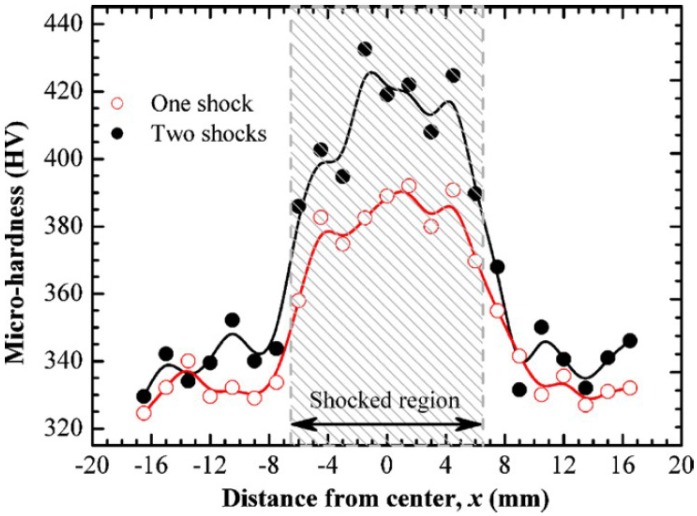
Microhardness at the surfaces of Ti-6Al-4V specimens shocked with three 40% overlapped spots [53].

**Figure 15:**
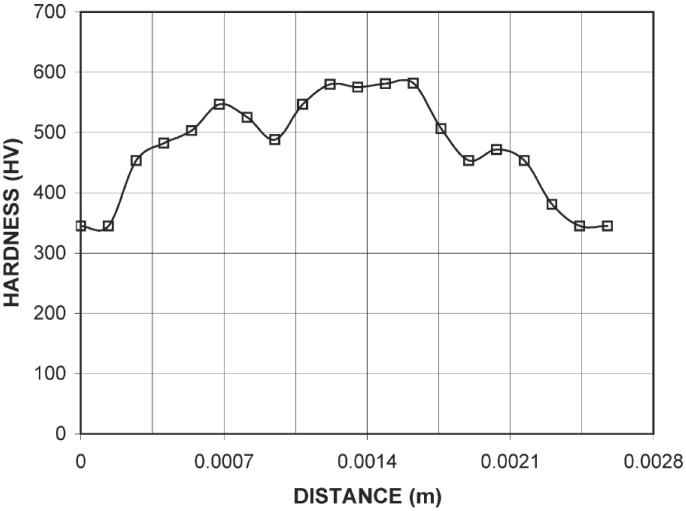
Microhardness across the irradiated surface of Ti-6Al-4V using a 300 gf load [93].

LSP process increases the surface hardness (nanohardness or microhardness) of the irradiated area [85,153,167] and this increase diminishes with depth into the material [96,117,134]. Surprisingly, no effect on hardness was observed on duplex stainless steel [86] which was attributed to the insufficient energy from the LSP. Therefore, hardness enhancement depends on the peening process such as power density [134,151], number of impacts [53,117], peak pressure [105,125] and substrate conditions. For instance, Zhang *et al.* [53] recorded a microhardness increase of 15% and 24% for one and two laser shocks on Ti-6Al-4V alloy with three 40% overlapped spots, as shown in the Figure 14. From this figure, it is evident that moving away from the shocked region results in hardness decrease to the base material, which has been attributed to the presence of tensile stresses [82]. Yilbas *et al.* [93] also found that the microhardness of the Ti-6Al-4V after LSP was 1.5 times of the base material as shown in Figure 15. The hardness increase was attributed to the increased dislocation densities [49,76,85,128] and twinning deformation [82,96]. In addition, a non-uniform pressure (load distribution) led to the variation of the hardness values across the surface, as shown in Figure 15. Table 5 shows a comparison between the hardness values obtained by [53] and [93].

**Table 5:** Comparison of microhardness (HV) values from Figure 14 and Figure 15.

Figure (Reference)	HV for one shock			HV for two shocks		
	Minimum	Maximum	ΔHV	Minimum	Maximum	ΔHV
Figure 15 [93]	350	580	230	-	-	-
Figure 14 [53]	360	395	35	385	425	40

Comparing Figure 14 and Figure 15 and Table 5, there is a difference in the hardness number for one and two shocks. For instance, ΔHV for one shock from [93] and [53] are 230 and 35, respectively. Furthermore, for the two shocks from [53], ΔHV is 40. However, it was reported that the difference between the maximum for one and two shocks was 30 [53]. Therefore, it can be seen from Figure 14 and the ΔHV data from [53] that the difference between the minimum and maximum values are relatively close as compared to the ΔHV data from [93]. The variations observed by Yilbas *et al.* [93] and Zhang *et al.* [53] could be due to the initial conditions of the treated sample or processing parameters. Similar trends were seen in laser peened Ti-17 [82], Al2024 [107], OFHC Cu [107], SUS 304SS [107], Al2011-T3 [62], Al6082-T651 [105]. Enhancement in nanohardness [96,111,126,137] after LSP was observed and attributed to dislocation multiplication and strain hardening. From comparative point of view, Mordyuk *et al.* [81] compared the effects of UIP and LSPwC and confining medium on microhardness of AISI 321. Microhardness of 3.9–4 GPa and 2.75 GPa for UIP and LSPwC were reported respectively [81]. The high microhardness from UIP can be attributed to the more plastic deformation and high compressive residual stresses induced compared to LSPwC. However, different hardness values might prevail when comparing UIP and LSP with coating.

Hardening of welded joints via LSP revealed enhancement in AA2195 [60,94] and Al7075 [61] which was attributed to the high dislocation densities and movements. Hardness of laser peened heat treated aluminum alloys (2024-T351, 2024-T851, 7075-T651, 7075-T73) was reported by Fairand and Clauer [5]. LSP showed hardness increase in 2024-T351 and no appreciable effect on peak aged 2024-T851 and 7075-T651 and over-aged 7075-T73.

**Figure 16:**
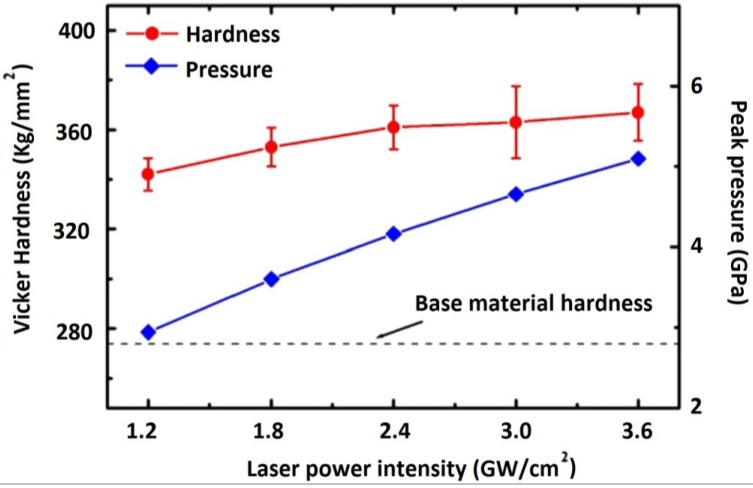
Surface hardness after warm laser shock peening (WLSP) at various laser power intensities and the corresponding peak plasma pressure [84].

Warm laser shock peening (WLSP) experiment on AISI 4140 by Liao *et al.* [84] showed that the hardness increased with increased power density and peak pressure as shown in Figure 16. It is evident from this figure that the highest hardness was at 2.4 GW/cm^2^ and 4 GPa after which, no appreciable increase was observed. The hardening effect was due to the effect of dislocation pinning induced by the second phase nanoprecipitates and strain hardening. Furthermore, they [84] stated that a post treatment (tempering) for 2 h showed further 28% enhancement in surface hardness while after 2 h, significant decrease in hardness was seen due to over tempering. This indicates that the introduction of temperature into the LSP process requires meticulous selection of LSP temperature and substrate conditions as well as post-shock tempering.

Similar to hardness, the yield strength is also affected by the LSP as investigated by Fairand and Clauer [5] on aluminum alloys (2024-T351, 2024-T851, 7075-T651, 7075-T73). They [5] reported yield strength increase in 7075-T73, less in 2024-T351 and no increase in 2024-T851 and 7075-T651. The possible reasons for no yield strength increase in 2024-T851 can be attributed to lack of tempering, good aging sequence and cooling mode as performed on 7075-T73. More so, no reason for the effect of LSP on 7075-T651 yield strength was highlighted by the authors [5] but it could be as a result of surface and in-depth compressive RS distribution as well as the inelastic strain from the shock wave [5]. However, more investigations are required for better understanding. Furthermore, LSP study on friction stir welded (FSW) joint of Al2195 showed that LSP after FSW increased the yield strength by 60% as compared to SP after FSW [104]. Similar work on welded 5086-H32 and 6061-T6 by [3] showed that the tensile yield strength of welded 5086-H32 raised up to the bulk level while that of 6061-T6 was between the welded and bulk levels. Again, LSP study on 00Cr12 stainless steel [118] further indicated yield strength enhancement. Thus, the increase in yield strength was attributed to the induced compressive residual stresses by LSP resulting in higher dislocations density.

Elastic modulus is another important material property which also depends on the LSP process conditions. For instance, LSP studies on 304SS [117], Fe-Ni Alloy [137] and LY2 Al [106] showed enhancement in elastic modulus while report by [62] on Al2011-T3 showed a decrease in elastic modulus after LSP. Due to rare reports in the literature relating the LSP parameters to elastic modulus, more fundamental understanding and experiments are still needed to explore this relationship and find out the responsible mechanism.

#### 3.4.2. Fatigue Life

**Figure 17:**
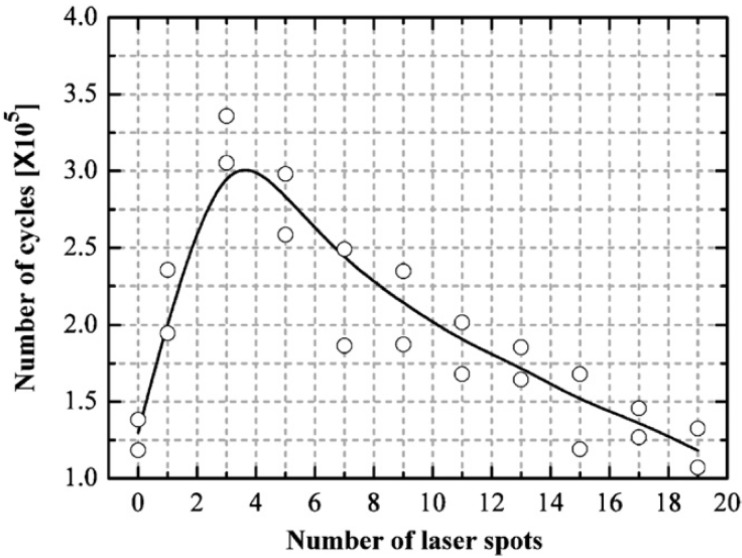
Variations of the fatigue lives with applied peak stress of 370 MPa along with the number of overlapped spots (40% overlap) [53].

**Figure 18:**
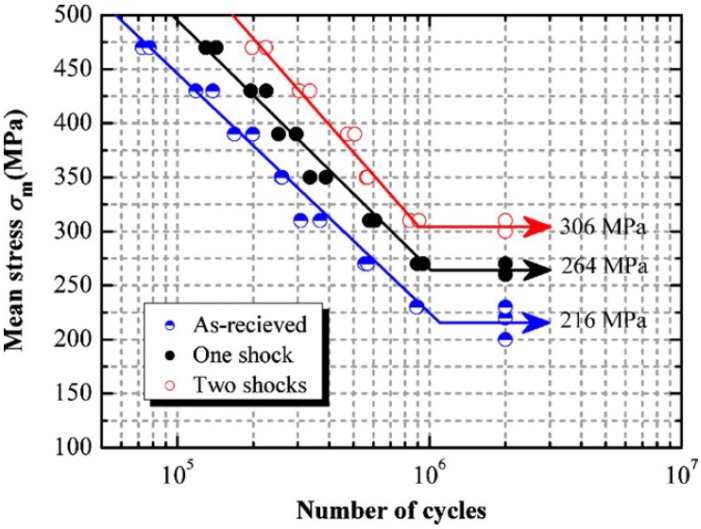
Fatigue lives of laser-peened and as-received Ti-6Al-4V at different applied stress levels [53].

LSP investigation on the fatigue life improvement of components was the main reason at the early stage of process development, where cracks are delayed and their propagation is retarded [6,11,55,56,152,168,169]. The higher depth of compressive residual stresses from LSP [11,75,87,88,90,100,104,112,149,170,171] made it a vital technique for such fatigue life improvement as compared to the conventional shot peening process. Furthermore, other contributing factors such as surface roughness [127] has been attributed to either the increase or decrease in fatigue life depending on the LSP processing conditions, such as the number of shocks [53]. Zhang *et al.* [53] investigated the fatigue life improvement of laser peened Ti-6Al-4V using one and two shocks with three 40% overlapped spots. For the fatigue testing, a load ratio of 0.3 was used. They [53] concluded that the fatigue life was increased and then decreased with increasing the number of shocks, as shown in Figure 17. For this test, 3 laser shocks gave the highest fatigue life. Comparing the peened with the as-received specimen, the fatigue life was increased by 22.2% and 41.7% for one and two shocks, respectively, as shown in Figure 18.

The reasons for the decrease in fatigue life in Figure 17 can be attributed to destruction of the absorbent layer, stress concentrations or increase in surface roughness due to the overlapped shocks, and instability of the compressive residual stresses [101]. It can also be said from Figure 18 that using the same applied stress level will result in still higher fatigue life for the laser peened specimens than what is shown in this figure.

**Figure 19:**
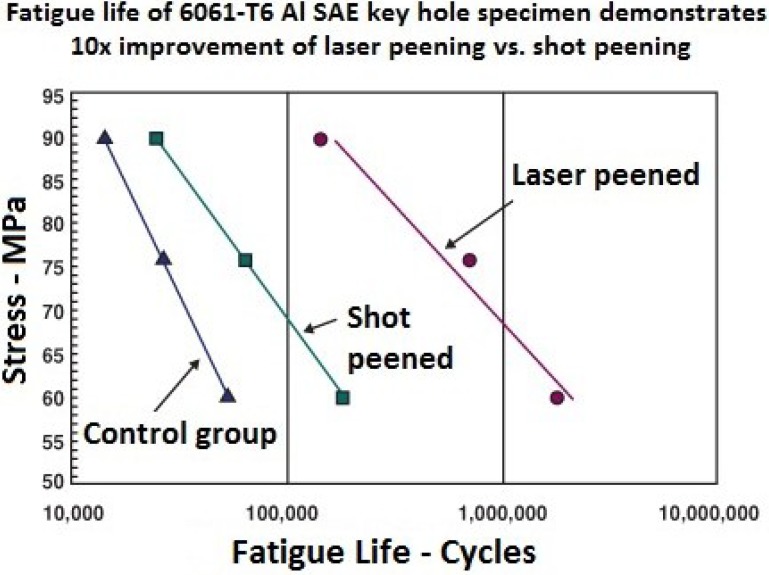
Fatigue life of 6061-T6 aluminum after LSP and SP [47].

Peyre *et al.* [76] reported that at high fatigue cycle testing, improvement in LSP sample was observed only at the crack initiation period as compared to SP and untreated specimens. Again, Metal Improvement Company (MIC) [47] studied the fatigue life of LSP and SP on Al6061-T6 key hole specimen. The fatigue life was improved 10 times after LSP in relation to SP specimen as shown in Figure 19. This improvement was attributed to the deeper layer of compressive residual stress with minimal cold work from the LSP process [150]. The deeper compressive stress layer might not be very beneficial in low cycle fatigue and at very high stress testing conditions.

One can therefore emphasize that the fatigue life testing depends on the conditions such as number of cycles, temperature and most importantly the stress levels. For instance, Nalla *et al.* [143] reported that fatigue life testing of Ti-6Al-4V at high stress and elevated temperature of 450 °C showed almost complete relaxation of the residual stresses. Ye *et al.* [101] attributed this relaxation to the instability of the compressive RS induced thereby having minimal benefit in extending fatigue life.

Experiments on bending fatigue limit of laser peened 55C1 steel coated with Al at stress ratio R = 0.1 showed 30% increase in the fatigue life [114]. The reason for this improvement was attributed to the induced compressive RS which was up to 80% of the yield strength of the material [114]. Further study on fatigue life of laser peened fillet and butt welded joints of SM490 [128] showed improvements in life for different stress ranges as compared to the unpeened welded joints. Similar enhancement was observed on welded 18Ni(250)SS [172]. The increased hardness and depth of the compressive RS at the joint were attributed to the enhancements in fatigue life.

Studies on the effect of LSP on the fatigue crack growth rate have been emphasized in the literature. For instance, Hatamleh [60] showed that the fatigue crack growth rate in stir welded AA2195 joint was greatly reduced by LSP as compared to the SP specimen. His work [60] further showed that the fatigue crack growth was comparable with the unwelded alloy. Yang *et al.* [102,103] reported the retardation in fatigue crack growth rate on Al2024-T3 for different notch configurations such as fastener hole, multiple crack stop holes and single-edge notches. Similar retardation in crack growth was observed in Al2024-T62 [75] and 2204 duplex SS [86]. The crack growth retardation can be attributed to the net effect of the compressive RS induced via LSP.

#### 3.4.3. Fretting Fatigue Life

Fretting fatigue life of contacting surfaces has received considerable attention and enhancements have been reported by surface treatments such as low plasticity burnishing [173], shot peening [86,173] and multilayer coatings [174]. However, only a handful of research investigation was made via LSP. For example, Srinivasan *et al.* [71] studied the fretting fatigue of laser peened Ti-6Al-4V with conditions similar to blade/disk contacts in gas turbines. An increase in fatigue life was reported as 5, 10 and 15 folds compared to the untreated samples. Similar fretting fatigue enhancement was seen by Liu and Hill [96] and King *et al.* [175] on Ti-6Al-4V. The induced compressive stresses were credited for the reported improvement in the fretting fatigue life. However, increase in hardness and better surface finish after LSP processing could be other reasons for the fretting fatigue improvement. Therefore, since surface roughness has significant effects during wear [115], fatigue and fretting fatigue life testing, the quality of the contact surface needs more attention. Furthermore, the study on the contact surface behavior can further be correlated to the combination of percentage overlap, number of shocks and techniques to eliminate stress raisers such as cracks from the LSP process. The process parameters optimization is essential because so far most researchers tried to improve the fretting fatigue life without meticulous attention to the process optimization.

### 3.5. Corrosion and Stress Corrosion Cracking (SCC) Behavior

#### 3.5.1. Corrosion Resistance Behavior

Due to the effectiveness of the induced compressive residual stresses by LSP in refining the microstructure, the corrosion behavior of materials can be improved. Studies have shown that this behavior depends on the LSP process, substrate conditions and environment. For instance, potentiodynamic polarization experiment by Kalainathan *et al.* [85] on laser peened 316L SS showed enhancement in corrosion potential and current density with increase in laser pulse density. Amar *et al.* [108] studied the corrosion behavior of laser peened AA2050-T8 using the scanning vibrating electrode technique. Their [108] LSP sample showed no intergranular corrosion, increased pitting potential and lower anodic current. Experiments using cyclic polarisation and EIS methods by Urdan *et al.* [102] showed enhanced passivity and reduced current in 0.6 M NaCl solution by factor of 12 on peened AA6082-T651 compared to unpeened sample. Peyre *et al.* [8] reported similar reduction of passivation current density and anodic shifts of the pitting potential of 316L SS in saline environment. They [8] attributed the resistance to the reduced pit formation as a result of the compressive RS. Berthe *et al.* [124] further emphasized that LSP treated G41400 showed corrosion current reduction in the martensitic structure. Thus, it can be said that there is a general consensus that LSP is an important technique for improving the corrosion resistance behavior of materials by reducing the anodic corrosion current. This can be attributed to the refinement of the grains and the effect of the compressive RS layer.

#### 3.5.2. Stress Corrosion Cracking (SCC)

**Figure 20:**
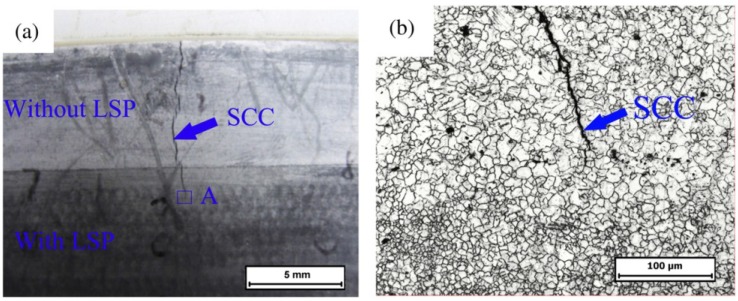
Stress corrosion cracking test results of AZ31B Mg alloy. (**a**) A photograph of Stress Corrosion Cracking (SCC) specimen; and (**b**) the microstructure of the rectangular region A in (**a**) [90].

Stress corrosion cracking (SCC) is the growth of crack as a result of aggressive environment and tensile stress. Mitigating this phenomenon using LSP is now gaining more attention due to the induced compressive RS which results in grain refinement and retardation of cracks [90]. More so, these translate to reduced passivation current density for instance, Scherpereel *et al.* [176] investigated the SCC behavior of two laser peened stainless steel grades (austenitic and martensitic) in 0.01 M NaCl+0.01 M Na_2_SO_4_ solution. The LSP samples showed improved SCC resistance by reducing the passivation current density without affecting the pitting potentials. Furthermore, studies on laser peened 304SS and Ni alloy [122] showed resistance to the SCC. Zhang *et al.* [90] studied the susceptibility of laser peened Mg alloy (AZ31B) to SCC. Specimens were laser peened with 50% overlap and the SCC test was conducted in a 1 wt % NaOH solution for 500 h at room temperature. Crack propagation was retarded in the peened section compared to the untreated section as shown in the Figure 20a,b shows an optical macrograph of region A from Figure 20a where the crack propagation was retarded. This was attributed to the refined microstructure after LSP.

**Figure 21:**
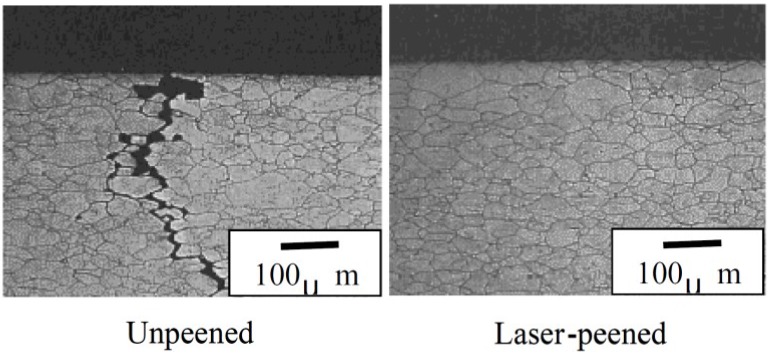
Microstructure of the cross section of the SCC tested 304SS samples [177].

Yoda and Newton [177] investigated the SCC susceptibility of peened and unpeened type 304SS by creviced bent beam (CBB) type tests. Their [177] results showed that all unpeened samples had cracks and propagations while peened samples showed no stress corrosion cracks [152] as indicated in Figure 21. Based on Figure 20 and Figure 21, it is evident that LSP can be a solution against SCC for different materials due to the induced compressive residual stresses [178,179].

### 3.6. Residual Stress Relaxation and Stability

**Figure 22:**
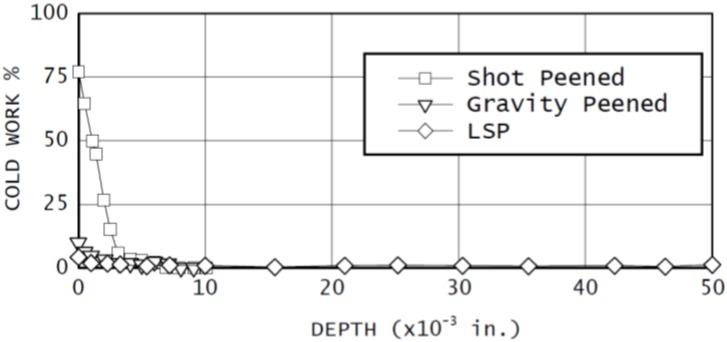
Cold work developed in Ti-6Al-4V after three peening processes [180].

The relaxation of residual stress has been a concern especially in compressor blades which are subjected to mechanical distortion [180]. This can be either a direct consequence of redistribution or relaxation of the induced stresses. It is worth mentioning here that three mechanisms were highlighted as primary causes of stress relaxation [180] viz; (1) compressive or tensile over loading; (2) cyclic loading near or above the endurance limit and (3) thermal cycling exposure. For instance, it has been observed that testing at very high stress level relaxes the stress induced by LSP [47]. Also, the work by Nikitin *et al.* [181] on AISI 304 showed that increase in temperature led to more stress relaxation. Furthermore, comparison of residual stress relaxation between LSP and SP shows that the low level of cold working during LSP process reduces this relaxation. However, this phenomenon depends on temperature and other processing parameters as investigated and modeled by Zhou *et al.* [65]. Prevey *et al.* [180] studied the thermal relaxation of induced compressive RS via SP and LSP in Ti-6Al-4V and IN718 alloys at elevated temperatures. Figure 22 shows the percentage of cold work induced by the SP and LSP process on Ti-6Al-4V. From this figure, it is evident that the SP induced a 75% cold work compared with only about 5% from LSP. At temperatures in the 400–500 °C range, about half of the compressive layer was relaxed in the highly cold worked surface from SP for both Ti-6Al-4V and IN718 alloys [180]. The work [180] also emphasized that the stress relaxation was directly proportional to the higher dislocation density and internal energy of the material. However, laser peened Ti-6Al-4V showed only a little relaxation at 475 °C and no relaxation for laser peened IN718 at 670 °C [180]. This was mainly attributed to the low level of cold working from LSP process [180]. More so, due to the lack of significant depth in residual stresses induced by UIP in comparison with LSP, the stress relaxation will be ultimately higher in UIP than LSP. This aspect has not been explored and this requires further understanding.

Interestingly, works by Ye *et al.* [69,91,105,182] showed that the stress relaxation can further be reduced in the LSP specimen if specimens were peened at elevated temperatures. This approach has shown stabilized compressive residual stresses by locking of mobile dislocations [69,91,105]. Therefore, careful selection of LSP temperature, pre-treatment and tempering temperatures as well as other processing parameters should be studied meticulously.

The stability of the induced residual stresses can also be achieved by combining the effect of laser sintering (LS) and LSP. This method has been employed by Lin *et al.* [183] on AISI 4140 where LS formed superhard nanoparticles near the top surface while LSP created an interaction between the nanoparticles and shock waves. The residual stress stability was realized through pinning of mobile dislocations similar to the stability observed by [69,91,105,182]. From their [183] work, the LS + LSP showed better fatigue performance as compared to LSP treated sample. This was attributed to the increased stress and strength stability. It will be interesting to compare the stress stability of AISI 4140 after LS + LSP and WLSP as both processes have been explored in [183] and [84] respectively.

## 4. Applications of Laser Shock Peening: Present and Future Status

Since inception, LSP has shown great potential and applicability at the industrial level especially for critical components. The technique has significantly improved the mechanical properties and performance of many engineering materials due to the increase in compressive RS depth and process control as discussed in Section 3.4 to Section 3.5. Several patents were issued and still increasing in number, for instance, GE Company was credited with 23 US patents between 1996 and 2001 [6]. Due to the advancement in the LSP technology, service providers such as Metal Improvement Company (MIC) [47] have now the capability to laser peen on- and off-site components [177] due to the advanced methods of laser beam delivery which included fixed beam for moving parts, moving beam for stationary part and scanning beam for large panels [48]. Furthermore, size of components ranging from small fuel injector to a hundred inch long wing panel can be successfully laser peened [48].

Industries such as aerospace have embraced the LSP for both commercial and military aircraft engines to mitigate fatigue failures. For instance, LSP has been applied to military aircraft engines such as F101-102-B-1B bomber, F110-129, F110-100, F110-132–F-16 fighter, F119-F-22 fighter, F118-100-B-2 bomber, F414-F/A-18E/F hornet and commercial engines such as CFM 56-B737, A320 Trent 500–Airbus, 340 Trent 800–Boeing 777, Trent 1000–Boeing 787, BR710–Gulfstream 500/550 BR725–Gulfstream 650 [184]. Laser peening of gas and steam turbine blades in power generation sector has also become a common practice [184]. Since 1991, Toshiba adapted underwater LSP in its nuclear power plant to peen 8 Japanese boiling water reactors (BWRs) and 2 Japanese pressure water reactors (PWRs) [177]. Potential application of LSP in biomedical implant components could be a feasible approach as demonstrated for Mg-Ca alloys [79] and proposed work by Kamkarrad *et al.* [185]. However, the compatibility of body tissues and induced compressive layers will require more understanding through research.

**Figure 23:**
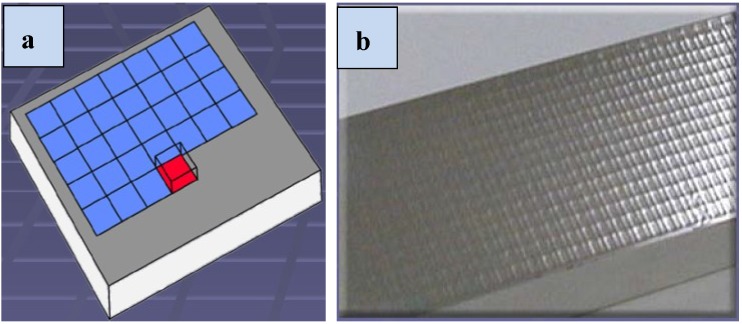
Square shaped beam pattern (**a**) and a peened component (**b**) [47].

Recent technological development in laser beam is the use of squared shaped laser beam. This approach has shown efficient coverage, overlapping, uniform packing and improved surface quality for a layer of treatment as compared to the round shaped beams [6,13,48,72,114,130]. Figure 23a,b show a typical square shaped beam pattern as well as a peened component respectively [47]. Geometrical limitations such as fillets, notches, shape edges could be challenging as most of reported works were on simple flat shaped geometries. More understanding in the aspect of beam shape and geometry is still required.

Researchers have successfully addressed the simulation of LSP process in a number of approaches. For instance, laser-material interactions to predict the pressure pulse magnitude and shape, residual stress profiles/distribution [66,72,88,186,187,188,189,190,191,192], laser non-conduction-limited heating [93], plastic strain [93], residual elastic strain [193], microcracking, surface deformation [158,194], material behavior near water-coating interface [195], powder compaction [184], and dislocation behavior [63] have been studied extensively. Fatigue crack propagation, thermal relaxation of residual stresses [65] and indentation profiles on the substrate [66] have been successfully modeled. The numerical models and experimental works have shown good agreements [131,196] especially for thick sections though little discrepancies might be identified. However, for thin sections, the modeling might be difficult due to the cyclic plastic deformation at the section center caused by complex wave pattern.

LSP on welded joints has shown enhancements in the hardness [61], yield strength [3,94,141], crack growth rate resistance [60] and fatigue life with a patent issued to Mannava *et al.* [46]. Further work by Sakino *et al.* [128] showed significant improvement in fatigue life [172] of laser peened fillet and butt welded joints of SM490 steel. Similar observations have been seen in UIP treated joints [19,31,32]. This indicates that compressive residual stresses are beneficial to welded joints irrespective of the technique.

Warm laser shock peening (WLSP) is another new emerging technology integrating the merits from the conventional LSP process, dynamic strain aging (DSA) and dynamic precipitation (DP) to enhance material performance. The WLSP process is carried out at a relatively high temperature. The selection of peening temperature depends on the material and the confining medium. Recently, Cheng’s group at Purdue University, USA applied the WLSP on Al6061-T6 [101] at 160 °C and 4140 steel at 250–300 °C [84] to investigate the effect of WLSP on fatigue behavior. The report [105,182] suggests that the WLSP showed better fatigue properties than the conventional LSP due to higher surface strength, deeper compressive residual stress and higher stability of dislocations. The improvements were attributed to the improved cyclic stability of compressive residual stress and locking of mobile dislocations due to DSA and DP. This observation is in accord with the work of Ren *et al.* [197] on laser peened ASTM:410L 00Cr12. More research studies on WLSP will further augment their [91,105] efforts in establishing this new approach.

Unlike WLSP, LSP can also be performed at a lower temperature in what is known as cryogenic laser shock peening (CLSP). This promising method produces severe plastic deformation (SPD) that generates nanotwinned microstructure thereby enhancing the properties. For instance, Ye *et al.* [198] explored this technique on copper in a liquid nitrogen (LN_2_) tank. They [198] showed increase in hardness and strength while preserving the ductility. This was attributed to the high dislocation density, nanograins and nanotwins from the CLSP. However, the CLSP will require more experimental investigations with regards to the cooling medium and target material. For example, the (LN_2_) used by Ye *et al.* [198] might have adverse effect on other materials. More so, in comparing CLSP with WLSP, materials processed using WLSP will show better properties due to the inherent enhancement by heat treatment.

**Figure 24:**
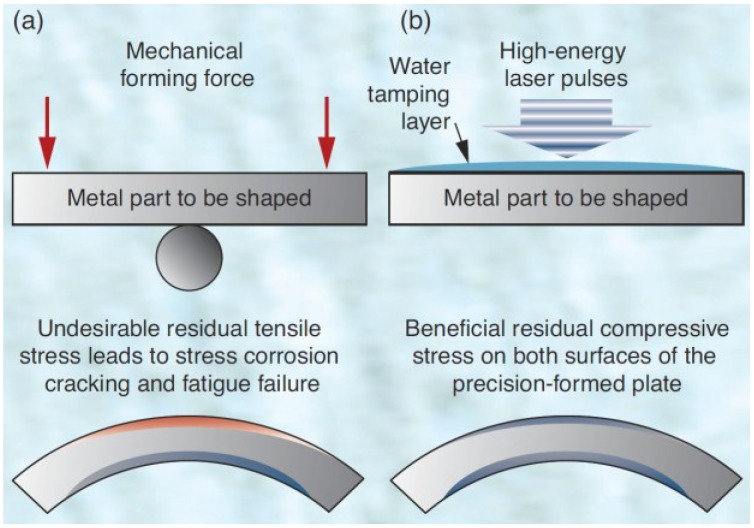
Comparison of (**a**) conventional and (**b**) Laser shot precision metal forming process [59].

**Figure 25:**
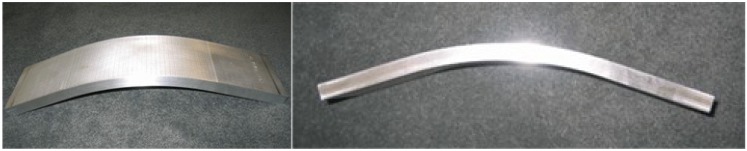
A 0.4 inch (10 mm) thick piece of Aluminum 7050 formed by laser peening [47].

LSP technique has showed the feasibility of shaping and forming of metal components using high energy laser pulses. This allows forming of thicker sections and tighter curves due to the deep compressive residual stresses as compared to other techniques such as shot peening [47,59]. Due to the induced stresses, elongation of the peened region occurs and consequently bending the overall shape [59]. Unlike the conventional mechanical forming, the forming force induces undesirable tensile residual stresses which may lead to stress corrosion cracking and fatigue failure [59] as shown in Figure 24. To demonstrate this, Metal Improvement Company formed a 10 mm thick Al7050 by laser peening and a radius of about 230 mm was achieved as shown in Figure 25. Similar forming processes have been studied in Cu [133], and Fe78Si9B13 [138]. However, more research studies are still required to simulate real working conditions such as high temperature applications where stresses might be relaxed.

**Figure 26:**
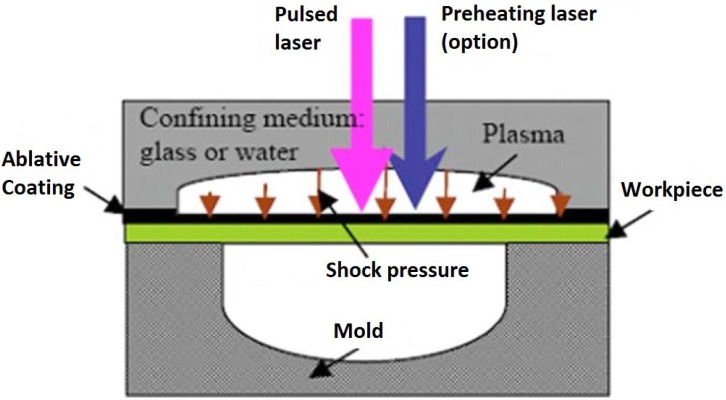
Schematic setup of the laser dynamic forming process [87].

Furthermore, Cheng *et al.* [87] used the laser dynamic forming (LDF) approach to form Cu foils. The LDF is a hybrid forming process that integrates the advantages of LSP and metal forming. Typical set-up of the process is depicted in Figure 26. Studying the substructure using electron backscatter diffraction (EBSD), they [87] found that high dislocation density and refined grains dominated the microstructure after LDF. This improved the strength of the components.

**Figure 27:**
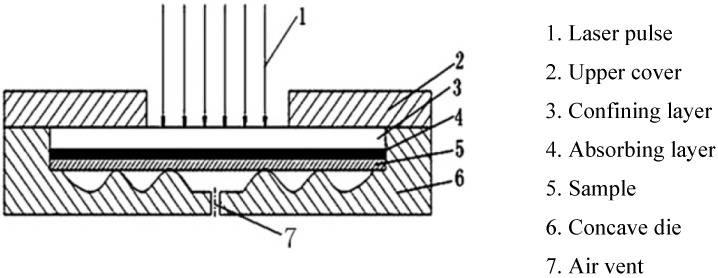
Schematic set-up of metal sheet laser shock forming with concave die [70].

**Figure 28:**
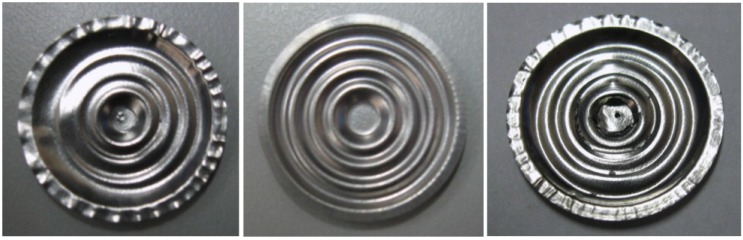
Appearance of semi die formed samples using 12 J (L), 15 J (M) and 18 J (R) [70].

Yu *et al.* [199] further studied the LDF of laminated composites on patterned 3-D surface. They [199] reported that the polymers absorbed the shock energy and had no adverse effect on the composite structure. Further investigation into the effect of surface defects and irregularities, variation in mechanical properties, and substrate conditions are essential for further improvement of the LDF technique. De-shaping after relaxation of stress could pose potential challenges in this new approach. Furthermore, the precision of the technique could be alarming as different forming and LSP process parameters could result in different patterns. For example, Jiang *et al.* [70] studied the precision control of metal sheet forming using 3J53 Ni alloy in a semi concave die with the typical set-up shown in Figure 27. Laser shock wave mechanical effect was used to plastically deform the metal sheet. The Nd:Glass laser type process parameters used were 10–40 J, 22 ns, and 6 mm spot diameter. Black paint and K9 glass were used as absorbent and confining media respectively. Jiang *et al.* [70] showed that the optimal laser pulse energy was 15 J for precision control in semi die forming using laser shock wave as depicted in Figure 28. It is evident from this figure that for the different pulse energies used, the outline and matching of the concave die produced different features. The 15 J-experiment gave the best outline of the concave die. However, more fundamental understanding on how the process affects the mechanical properties and microstructure such as spring back effect, reverse deformation is still needed. Furthermore, extending the macroforming to microforming scale [87] will be an interesting area of research especially in applications such as bulge forming, welding and bending [184]. Since macroforming or microforming requires high precision control, the use of UIP technique might not be beneficial especially for complex geometries due to the handheld apparatus employed. Again, the use of SP technique might not be suitable due to the high level of cold working thereby leading to high stress relaxation. These reasons will entail having variation in dimensions, microstructure as well as properties.

Use of laser shock wave for adhesion testing of coatings is yet another interesting application. Bolis *et al.* [80] investigated the debonding mechanism of electroplated 70–90 µm non-porous and non-rough Ni coating deposited on a pure Cu substrate (120–190 µm). Simulation codes SHYLAC [51,81] and HUGO [80] were employed to further validate the result. Both the experimental and simulation works showed that the interface debonding and debonding fatigue can be determined. However, from application point of view, more studies are required in order to verify the efficiency and accuracy of the technique. Bossi *et al.* [200] also inspected the surface of composites and other joints using the laser bond inspection technique. They [200] elucidated promising results for the 23 mm thick samples but thicker samples pose challenges to the applicability of the technique. However, more investigations are needed for complex coatings such as multi layered coatings, process optimization and holistic quantitative evaluation of the adhesion.

While significant improvements on corrosion and stress corrosion cracking behavior of materials via LSP have been reported, no reports could be found in the literature on the improvement of water droplet erosion (WDE) resistance via LSP. However, attempts are being made by the authors of this paper at Concordia University, Canada to investigate the water erosion behavior of laser peened materials such as Ti-6Al-4V used in compressor blades of gas turbines. Preliminarily, the on-going research shows that the LSP process might be a potential solution in enhancing the WDE behavior of materials. The WDE is a special form of erosion with high velocity liquid droplets on a solid surface [201]. The mechanism of the impingement process is complex by virtue of the many parameters involved including: impact velocity, impact angle, droplet size and size distribution, droplet shape, frequency of impacts and condition of the tested material. Other laser surface treatments such as laser surface alloying and laser nitriding have shown improvement against water erosion. For instance, Robinson and Reed [202] used continuous wave CO_2_ laser to modify the surface of Ti-6Al-4V in both inert and dilute nitrogen atmospheres. An improvement of 10% in micro-hardness and homogeneity of the microstructure improved the water erosion resistance after 25 h erosion. A mass loss of the laser treated sample is about 20%–25% as compared to the untreated sample. Therefore, due to the unavailability of the LSP-WDE information, fundamental and experimental understanding on how the LSP process can improve this resistance is needed. This approach will drive to check which factors (hardness, depths of compressive residual stresses, multiple laser shots, and overlaps) affect the water erosion resistance significantly. Microstructural analysis can be instrumental in understanding the different stages of the erosion process such as incubation, acceleration, deceleration and terminal stages. The synergistic investigation can provide a more systematic and scientific explanation of the improvement against water erosion using LSP. Based on the aforementioned applications, one can argue that the LSP technique has a more promising future over the UIP and SP techniques. This can be attributed to the higher depth of compressive RS, low level of cold working, precision and repeatability of the process. However, in certain applications such as reduction of welding residual stress, UIP can be competitive with LSP due to lower cost and more flexibility as compared to the high cost of operation when employing LSP. This is one of the challenges and drawbacks of the LSP process that will be addressed in the following section.

## 5. Challenges for the LSP Process

So far the LSP technique has shown numerous advantages over the conventional SP, but one cannot ignore the drawbacks and shortcomings related to the technique. This section discusses some of the challenges pertinent to LSP.

LSP induces deep compressive RS which must be equally balanced with tensile stresses. This is a challenge especially when thin regions are peened and tensile stresses reside beneath the peened surface [98]. This phenomenon is usually observed when the compressive RS does not pass through the entire section thickness [203]. Possible solution is to peen both sides of the component which is mostly found in turbine airfoil applications [65,203,204]. In fact, this approach has been applied successfully in investigating the foreign object damage (FOD) of leading edge of turbine blades made of Ti-6Al-4V [11,83,205,206,207]. Another possibility is to constrain the tensile stresses to dwell in non-critical locations. General Electric Infrastructure-Aviation (GEA) applies mathematical design optimization to tackle this point [203].

Dimensional variation is another challenge posed by the LSP process which leads to distortion of the components. This could be related to the thickness, LSP processing and other geometrical constraints. For instance, studies have shown the lengthening of the leading edge in an airfoil in the radial direction due to compressive stresses while the other parts distorting as a result of tensile stresses [203,207]. GEA further demonstrated that for airfoils, less twisting was expected for the root due to the large thickness than at the tip. Further investigation on the effect of distortion of components will add much needed technical information necessary for the industry to adopt additional use of LSP.

The lack of non-destructive testing technology for quality control and quality assurance (QA/QC) is a major challenge for laser peened sections. This technology will be very useful for the LSP process validation. Presently, destructive testing such as high or low fatigue testing at different operating conditions are the only ways used to evaluate most materials [203] and to validate the suitability of LSP process. The harmonious agreement between the destructive and non-destructive testing will boost the confidence level of the process in many applications.

Due to the desire to have maximum magnitude of compressive RS, it has been reported that the LSP process could induce internal rupture of components due to over-processing [203]. Cracks of less than 0.002 inch wide normal to the stress wave propagation result from such over processing [82,87,118,203]. Process optimization and careful selection of the process parameters will be paramount to eliminate the possibility of having such cracks which can be a defect in critical components.

Despite the fact that LSP has shown less thermal relaxation of stresses at elevated temperatures compared to SP technique due to little cold working, the relaxation is profound in laser peened components at high temperatures exceeding 540 °C. This is a challenge in high pressure compressors where 15% reduction in fatigue life was reported for thermally cycled Ti alloys at temperature of >540 °C for over 200 h [203]. This could be attributed to the relaxed compressive stresses at such elevated temperature and exposure time [65,180]. However, recent research studies by [69,91,105] showed that peening at an elevated temperature reduced such relaxations due to stability of the compressive RS.

For every new technical concept, the cost of conducting research and applying it in industry is a major challenge and LSP is not an exception in this regard. LSP is a high-cost technology as compared to SP [150] and UIP especially when the component efficiency and in-service performance are very essential for the application [203]. For this reason, when considering high scale production, companies will find it difficult to laser peen fabricated components. However, the integration of LSP technology at a strategic point during the production line could help minimize the cost. More so, investigations using modeling might further minimize the experimental cost by reducing the need to do all the parametric combinations [82,208].

## 6. Summary

Laser peening process has been seen to induce compressive residual stresses reaching about 4–5 times deeper and higher intensity with uniformity across material surface than the conventional shot peening technique. However, the SP has not been replaced by the laser peening especially in applications were coatings are used. Some researchers are of the opinion that both techniques have their distinct merits and challenges. However, LSP has shown superior improvements in fatigue life, fretting fatigue life and SCC due to the refined microstructure and enhanced mechanical properties. These enhancements have been shown in commercially available structural materials such as low carbon steels, nickel based alloys, stainless steels, titanium alloys, magnesium alloys and aluminum alloys. Recent studies on laser forming, warm laser peening show that researchers have gone beyond the initial fatigue life improvement concept to a more widen approach, although more studies are required.

Despite the enhancements associated with the LSP technique, more research studies are still needed for complete understanding. For instance, the lack of standardized LSP process parameters is still an issue, however, the Society of Automotive Engineers had attempted to provide optimized process parameters for various materials but till date no such report is published. However, this could be due to the multi factors associated with the peening process such as the laser type, and peening and substrate (target) conditions.

With the numerous compressive residual stress measurement techniques such as X-ray diffraction, neutron diffraction, hole drilling, contour and slitting compliance methods, there are still discrepancies in quantifying the surface and in-depth residual stresses. This might be due to the extrinsic nature of the residual stresses measured from the strains in the materials and the associated correction methods that should be used. However, with more experimental and simulation works, accurate predictions of the residual stresses with special attention given to the geometrical constraints can be established.

In spite of the fact that medical implant materials can be laser peened with enhanced properties, there is still lack of experimental data to substantiate this fact. More research findings on the compatibility of laser treated surfaces with body tissues are still missing.

The introduction of temperature effect into the LSP process in what is known as warm or thermal engineered LSP has just emerged with few publications to date. This process integrates the advantages of the LSP with dynamic strain aging and dynamic precipitation. Surface properties and fatigue life of steel and aluminum have been enhanced due to dislocation pinning effect during the dynamic precipitation. Furthermore, WLSP has shown that, stress relaxation can be greatly reduced while enhancing the material properties and this can be a solution to the stress relaxation challenges posed by the conventional LSP. However, this can only be achieved with more fundamental understanding and experimental works on the careful choice of processing temperature and confining medium as water might not be applicable at elevated temperatures.

The feasibility of forming complex and curved geometries via the laser shock processing has shown that the conventional metal forming processes can potentially be replaced to some extent. This is due to the fact that the tensile stress leading to SCC is totally eliminated by inducing compressive residual stresses. However, only a few investigations have shown this approach and more research addressing issues such as temperature limitation, angle variations, spring back phenomenon are still needed. It is worth mentioning that the feasibility of forming using SP might be difficult due to high level of cold working which results in significant stress relaxation. More so, the use of UIP could also pose the difficulty of having dimensional accuracy due to the handheld apparatus employed in the process.

UIP process has also shown similar to improvements offered by LSP especially in the fatigue life enhancement of welded joints. However, only a few comparisons were made between the two processes. Notwithstanding, when considering the cost of operation and location of operation such as treatment in austere environment, UIP will be a better candidate than LSP due to the simplicity and transportability of the process and equipment respectively.

Remarkable improvements in corrosion and stress corrosion cracking behavior of materials via laser peening have been reported. This was attributed to the refined microstructure and induced compressive residual stresses. More so, this peening process is expected to enhance the water droplet erosion behavior of materials, this research has not been explored but attempts are being made by the authors of this paper. The experimental results and discussion from the work will be published in due course.

## References

[1] Fairand B.P., Clauer A.H. (1979). Laser generation of high-amplitude stress waves in materials. J. Appl. Phys..

[2] Fairand B.P., Wilcox B.A., Gallagher W.J., Williams D.N. (1972). Laser shock-induced microstructural and mechanical property changes in 7075 aluminum. J. Appl. Phys..

[3] Clauer A.H., Fairand B.P., Wilcox B.A. (1977). Laser shock hardening of weld zones in aluminum alloys. Metall. Trans. A.

[4] Clauer A.H., Fairand B.P., Wilcox B.A. (1977). Pulsed laser induced deformation in an Fe-3%wt Si alloy. Metall. Trans. A.

[5] Fairand A.H., Clauer B.P. (1979). Interaction of laser-induced stress waves with metals. Proceedings of the ASM Conference Applications of Lasers in Materials Processing.

[6] Montross C., Wei T., Ye L., Clark G., Mai Y. (2006). Laser shock processing and its effects on microstructure and properties of metal alloys: A review. Int. J. Fatigue.

[7] Peyre P., Fabbaro R. (1995). Laser shock processing: A review of the physics and applications. Opt. Quant. Electron..

[8] Peyre P., Scherpereel X., Berthe L., Carboni C., Fabbro R., Béranger G., Lemaitre C. (2000). Surface modifications induced in 316 L steel by laser peening and shot-peening. Influence on pitting corrosion resistance. Mater. Sci. Eng. A.

[9] Peyre R., Scherpereel P., Berthe X., Fabbro L. (1998). Current trends in laser shock processing. Surf. Eng..

[10] Clauer A. (2009). A historical perspective on laser shock peening. Met. Finish. News.

[11] Ruschau J., John R., Thompson S.R., Nicholas T. (1999). Fatigue crack nucleation and growth rate behavior of laser shock peened titanium. Int. J. Fatigue.

[12] Martinez S.A., Sathish S., Blodgett M.P., Shepard M.J. (2003). Residual stress distribution on surface-treated Ti-6AI-4V by X-ray diffraction. Exp. Mech..

[13] Cao Z., Xu H., Zou S., Che Z. (2012). Investigation of surface integrity on TC17 titanium alloy treated by square-spot laser shock peening. Chin. J. Aeronaut..

[14] Vaccari J. (1992). Laser shocking extends fatigue life. Am. Mach..

[15] Mannava S. (1998). On the Fly Laser Shock Peening-General Electric Company. U.S. Patent.

[16] Mordyuk B.N., Prokopenko G.I. (2006). Fatigue life improvement of α-titanium by novel ultrasonically assisted technique. Mater. Sci. Eng. A.

[17] Hou L., Wang D., Zhang Y. (2005). Investigation of the fatigue behaviour of the welded joints treated by TIG dressing and ultrasonic peening under variable-amplitude load. Int. J. Fatigue.

[18] Xing Y.M., Lu J. (2004). An experimental study of residual stress induced by ultrasonic shot peening. J. Mater. Process. Technol..

[19] Abdullah A., Malaki M., Eskandari A. (2012). Strength enhancement of the welded structures by ultrasonic peening. Mater. Des..

[20] Mordyuk B.N., Iefimov M.O., Prokopenko G.I., Golub T.V., Danylenko M.I. (2010). Structure, microhardness and damping characteristics of Al matrix composite reinforced with AlCuFe or Ti using ultrasonic impact peening. Surf. Coat. Technol..

[21] Abramov V.O., Abramov O.V., Sommer F., Gradov O.M., Smirnov O.M. (1998). Surface hardening of metals by ultrasonically accelerated small metal balls. Ultrasonics.

[22] Liu G., Lu J., Lu K. (2000). Surface nanocrystallization of 316 L stainless steel induced by ultrasonic shot peening. Mater. Sci. Eng. A.

[23] Sandá A., García Navas V., Gonzalo O. (2011). Surface state of Inconel 718 ultrasonic shot peened: Effect of processing time, material and quantity of shot balls and distance from radiating surface to sample. Mater. Des..

[24] Tao N., Sui M., Lu J., Lua K. (1999). Surface nanocrystallization of iron induced by ultrasonic shot peening. Nanostruct. Mater..

[25] Yin D., Wang D., Jing H., Huo L. (2010). The effects of ultrasonic peening treatment on the ultra-long life fatigue behavior of welded joints. Mater. Des..

[26] Mordyuk B.N., Prokopenko G.I. (2007). Ultrasonic impact peening for the surface properties’ managemen. J. Sound Vib..

[27] Liao M., Chen W.R., Bellinger N.C. (2008). Effects of ultrasonic impact treatment on fatigue behavior of naturally exfoliated aluminum alloys. Int. J. Fatigue.

[28] Roy S., Fisher J. (2005). Enhancing fatigue strength by ultrasonic impact treatment. Steel Struct..

[29] Vilhauer B., Bennett C.R., Matamoros A.B., Rolfe S.T. (2012). Fatigue behavior of welded coverplates treated with Ultrasonic Impact Treatment and bolting. Eng. Struct..

[30] Roy S. (2003). Fatigue resistance of welded details enhanced by ultrasonic impact treatment (UIT). Int. J. Fatigue.

[31] Berg-Pollack A., Voellmecke F.J., Sonsino C.M. (2011). Fatigue strength improvement by ultrasonic impact treatment of highly stressed spokes of cast aluminium wheels. Int. J. Fatigue.

[32] Lihavainen V.M., Marquis G., Statnikov E.S. (2004). Fatigue strength of a longitudinal attachment improved by ultrasonic impact treatment. Weld. World.

[33] Yang X., Ling X., Zhou J. (2014). Optimization of the fatigue resistance of AISI304 stainless steel by ultrasonic impact treatment. Int. J. Fatigue.

[34] Statnikov E.S., Korolkov O.V., Vityazev V.N. (2006). Physics and mechanism of ultrasonic impact. Ultrasonics.

[35] Statnikov E. (2004). Physics and mechanism of ultrasonic impact treatment. Int. Inst. Weld..

[36] An X., Rodopoulos C.A., Statnikov E.S., Vitazev V.N., Korolkov O.V. (2006). Study of the surface nanocrystallization induced by the esonix ultrasonic impact treatment on the near-surface of 2024-T351 aluminum alloy. J. Mater. Eng. Perform..

[37] Yang X., Zhou J., Ling X. (2012). Study on plastic damage of AISI 304 stainless steel induced by ultrasonic impact treatment. Mater. Des..

[38] Fan Z., Xu H., Li D., Zhang L., Liao L. (2012). Surface nanocrystallization of 35# type carbon steel induced by ultrasonic impact treatment (UIT). Procedia Eng..

[39] Galtier A., Statnikov E.S. (2004). The influence of ultrasonic impact treatment on fatigue behaviour of welded joints in high-strength steel. Weld. World.

[40] Günther H., Kuhlmann U., Durr A. (2005). Rehabilitation of welded joints by ultrasonic impact treatment (UIT). Proceedings of the IABSE Symposium Report.

[41] LSP Technologies Surface enhancement technologies. http://www.lsptechnologies.com.

[42] Mannava S., Cowie W.D. (1996). Technique to Prevent or Divert Cracks-General Electric Company. U.S. Patent.

[43] Luo K.Y., Wang C.Y., Li Y.M., Luo M., Huang S., Hua X.J., Lu J.Z. (2014). Effects of laser shock peening and groove spacing on the wear behavior of non-smooth surface fabricated by laser surface texturing. Appl. Surf. Sci..

[44] Dane C., Hackel L., Daly J., Harirson J. (1997). Laser peening of metals enabling laser technology. Adv. Mater. Process..

[45] Mannava S., Ferrigno S. (1997). Laser Shock Peening for Gas Turbine Engine Vane Repair-General Electric Company. U.S. Patent.

[46] Mannava S., Ferrigno S., Cowie W.D. (1998). Laser Shock Peening for Gas Turbine Engine Weld Repair-General Electric Company. U.S. Patent.

[47] Metal Improvement Company: Shot peening. http://www.metalimprovement.com/.

[48] Dane C.B., Harris F., Lao E., Rankin J., Hurd R. (2010). Advanced beam delivery for mobile laser peening. Proceedings of the 2nd International Conference on Laser Peening.

[49] Clauer A.H., Holbrook J.H., Fairand B.P. (1981). Effects of laser induced shock waves on metals. Shock Waves and High Strain Rate Phenomena in Metals.

[50] Clauer A.H. (1996). Laser shock of peening for fatigue resistance. Surface Performance of Titanium.

[51] Fabbro R., Peyre P., Berthe L., Scherpereel X. (1998). Physics and applications of laser-shock processing. J. Laser Appl..

[52] Kruusing A. (2004). Underwater and water-assisted laser processing: Part 1-general features, steam cleaning and shock processing. Opt. Lasers Eng..

[53] Zhang X.C., Zhang Y.K., Lu J.Z., Xuan F.Z., Wang Z.D., Tu S.T. (2010). Improvement of fatigue life of Ti-6Al-4V alloy by laser shock peening. Mater. Sci. Eng. A.

[54] Guo Y., Caslaru R. (2011). Fabrication and characterization of micro dent arrays produced by laser shock peening on titanium Ti-6Al-4V surfaces. J. Mater. Process. Technol..

[55] Brar N.S., Hopkins A., Laber M.W. (2000). Laser shock peening of titanium 6–4 alloy. AIP Conf. Proc..

[56] Tani G., Orazi L., Fortunato A., Ascari A., Campana G. (2011). Warm laser shock peening—New developments and process optimization. CIRP Ann. Manuf. Tech..

[57] Rubio-González C., Gomez-Rosas G., Ocaña J., Molpeceres C., Banderas A., Porro J., Morales M. (2006). Effect of an absorbent overlay on the residual stress field induced by laser shock processing on aluminum samples. Appl. Surf. Sci..

[58] Golden J., Hutson A., Sundaram V., Arps J. (2007). Effect of surface treatments on fretting fatigue of Ti-6Al-4V. Int. J. Fatigue.

[59] Chen H., Claudet A., Zaleski T., Dane C., Lane L., Hackel L., Harris F., Halpin J. (2003). Laser process form thick, curved metal parts. Sci. Technol. Rev..

[60] Hatamleh O. (2009). A comprehensive investigation on the effects of laser and shot peening on fatigue crack growth in friction stir welded AA 2195 joints. Int. J. Fatigue.

[61] Hatamleh O., DeWald A. (2009). An investigation of the peening effects on the residual stresses in friction stir welded 2195 and 7075 aluminum alloy joints. J. Mater. Process. Technol..

[62] Montross C., Florea V., Swain M. (2011). The influence of coatings on subsurface mechanical properties of laser peened 2011-T3 aluminum. J. Mater. Sci..

[63] Cheng G.J., Shehadeh M.A. (2005). Dislocation behavior in silicon crystal induced by laser shock peening-A multiscale simulation approach. Scripta Mater..

[64] Hong X., Wang S., Guo D., Wu H., Wang J., Dai Y., Xia X., Xie Y. (1998). Confining medium and absorptive overlay: Their effects on a laser-induced shock wave. Opt. Lasers Eng..

[65] Zhou Z., Bhamare S., Ramakrishnan G., Mannava S.R., Langer K., Wen Y., Qian D., Vasudevan V.K. (2012). Thermal relaxation of residual stress in laser shock peened Ti-6Al-4V alloy. Surf. Coat. Technol..

[66] Cao Y., Shin Y., Wu B. (2010). Parametric study on single shot and overlapping laser shock peening on various metals via modeling and experiments. J. Manuf. Sci. Eng..

[67] Thareja R., Shukla S. (2007). Synthesis and characterization of zinc oxide nanoparticles by laser ablation of zinc in liquid. Appl. Surf. Sci..

[68] Ling X., Peng W., Ma G. (2008). Influence of laser peening parameters on residual stress field of 304 stainless steel. J. Press. Vessel Technol..

[69] Ye C., Suslov S., Kim B.J., Stach E.A., Cheng G.J. (2011). Fatigue performance improvement in AISI 4140 steel by dynamic strain aging and dynamic precipitation during warm laser shock peening. Acta Mater..

[70] Jiang Y., Huang Y., Jin H., Gu Y., Ren A., Huang L., Qian X. (2013). Research on precision control of sheet metal forming by laser shock waves with semi-die. Opt. Lasers Eng..

[71] Srinivasan S., Garcia D.B., Gean M.C., Murthy H., Farris T.N. (2009). Fretting fatigue of laser shock peened Ti-6Al-4V. Tribol. Int..

[72] Cao Z., Che Z., Zou S., Fei Q. (2011). Numerical simulation of residual stress field induced by laser shock processing with square spot. J. Shanghai Univ..

[73] Kim J.H., Kim Y.J., Kim J.S. (2012). Effects of laser source geometry on laser shock peening residual stress. Korean Soc. Mech. Eng..

[74] Rhode R., Johnson J. (1971). Dynamic deformation twinning in shock loaded iron. J. Appl. Phys..

[75] Hong Z., Chengye Y. (1998). Laser shock processing of 2024-T62 aluminum alloy. Mater. Sci. Eng. A.

[76] Peyre P., Fabbro R., Merrien P., Lieurade H. (1996). Laser shock processing of aluminium alloys. Application to high cycle fatigue behaviour. Mater. Sci. Eng. A.

[77] Yang C., Hodgson P.D., Liu Q., Ye L. (2008). Geometrical effects on residual stresses in 7050-T7451 aluminum alloy rods subject to laser shock peening. J. Mater. Process. Technol..

[78] Laser Research Center, Lidaris Ltd, Vilnius University. http://lidaris.com/glossary-2/fluence/.

[79] Sealy M.P., Guo Y.B. (2010). Surface integrity and process mechanics of laser shock peening of novel biodegradable magnesium-calcium (Mg-Ca) alloy. J. Mech. Behav. Biomed. Mater..

[80] Bolis C., Berthe L., Boustie M., Arrigoni M., Barradas S., Jeandin M. (2007). Physical approach to adhesion testing using laser-driven shock waves. J. Phys. D Appl. Phys..

[81] Mordyuk B.N., Milman Y.V., Iefimov M.O., Prokopenko G.I., Silberschmidt V.V., Danylenko M.I., Kotko A.V. (2008). Characterization of ultrasonically peened and laser shock peened surface layers of AISI 321 stainless steel. Surf. Coat. Technol..

[82] Cellard C., Retraint D., François M., Rouhaud E., le Saunier D. (2012). Laser shock peening of Ti-17 titanium alloy: Influence of process parameters. Mater. Sci. Eng. A.

[83] Tan Y., Wu G., Yang J., Pan T. (2004). Laser shock peening on fatigue crack growth behaviour. Fatigue Fract. Eng. Mater. Struct..

[84] Liao Y., Suslov S., Ye C., Cheng G. (2012). The mechanisms of thermal engineered laser shock peening for enhanced fatigue performance. Acta Mater..

[85] Kalainathan S., Sathyajith S., Swaroop S. (2012). Effect of laser shot peening without coating on the surface properties and corrosion behavior of 316 L steel. Opt. Lasers Eng..

[86] Rubio-González C., Felix-Martinez C., Gomez-Rosas G., Ocaña J.L., Morales M., Porro J.A. (2011). Effect of laser shock processing on fatigue crack growth of duplex stainless steel. Mater. Sci. Eng. A.

[87] Cheng G.J., Pirzada D., Ming Z. (2007). Microstructure and mechanical property characterizations of metal foil after microscale laser dynamic forming. J. Appl. Phys..

[88] Hill M.R., Dewald A.T., Rankin J.E., Lee M.J. (2005). Measurement of laser peening residual stresses. J. Mater. Sci. Technol..

[89] Bugayev A.A., Gupta M.C., Payne R. (2006). Laser processing of inconel 600 and surface structure. Opt. Lasers Eng..

[90] Zhang Y., You J., Lu J., Cui C., Jiang Y., Ren X. (2010). Effects of laser shock processing on stress corrosion cracking susceptibility of AZ31B magnesium alloy. Surf. Coat. Technol..

[91] Wang F., Yao Z., Deng Q. (2007). Experimental study on laser shock processing of brass. J. Univ. Sci. Technol. Beijing Miner. Metall. Mater..

[92] Forget P., Strudel J.L., Jeandin M., Lu J., Castex L. (1990). Laser shock surface treatment of Ni-based superalloys. Mater. Manuf. Process..

[93] Yilbas B.S., Gondal M.A., Arif A.M., Shuja S.Z. (2004). Laser shock processing of Ti-6Al-4V alloy. Proc. Instn. Mech. Eng..

[94] Zabeen S., Preuss M., Withers P.J. (2013). Residual stresses caused by head-on and 45° foreign object damage for a laser shock peened Ti-6Al-4V alloy aerofoil. Mater. Sci. Eng. A.

[95] Rozmus-Górnikowska M. (2010). Surface modifications of a Ti-6Al-4V alloy by a laser shock processing. Acta Phys. Pol. A.

[96] Liu K.K., Hill M.R. (2009). The effects of laser peening and shot peening on fretting fatigue in Ti-6Al-4V coupons. Tribol. Int..

[97] Altenberger I., Nalla R.K., Sano Y., Wagner L., Ritchie R.O. (2012). On the effect of deep-rolling and laser-peening on the stress-controlled low- and high-cycle fatigue behavior of Ti-6Al-4V at elevated temperatures up to 550 °C. Int. J. Fatigue.

[98] Brockman R.A., Braisted W.R., Olson S.E., Tenaglia R.D., Clauer A.H., Langer K., Shepard M.J. (2012). Prediction and characterization of residual stresses from laser shock peening. Int. J. Fatigue.

[99] Dane C., Hackel L., Daly J. (1998). Shot peening with laser. Adv. Mater. Process..

[100] Wagner L., Mhaede M., Wollmann M., Altenberger I., Sano Y. (2011). Surface layer properties and fatigue behavior in Al 7075-T73 and Ti-6Al-4V: Comparing results after laser peening, shot peening and ball-burnishing. Int. J. Struct. Integr..

[101] Ye C., Liao Y., Cheng G.J. (2010). Warm laser shock peening driven nanostructures and their effects on fatigue performance in aluminium alloy 6160. Adv. Eng. Mater..

[102] Trdan U., Grum J. (2012). Evaluation of corrosion resistance of AA6082-T651 aluminium alloy after laser shock peening by means of cyclic polarisation and ElS methods. Corros. Sci..

[103] Ocaña J.L., Molpeceres C., Porro J.A., Gómez G., Morales M. (2004). Experimental assessment of the influence of irradiation parameters on surface deformation and residual stresses in laser shock processed metallic alloys. Appl. Surf. Sci..

[104] Hatamleh O. (2008). Effects of peening on mechanical properties in friction stir welded 2195 aluminum alloy joints. Mater. Sci. Eng. A.

[105] Trdan U., Porro J.A., Ocaña J.L., Grum J. (2012). Laser shock peening without absorbent coating (LSPwC) effect on 3D surface topography and mechanical properties of 6082-T651 Al alloy. Surf. Coat. Technol..

[106] Luo K., Lu J., Zhang L., Zhong J., Guan H., Qian X. (2010). The microstructural mechanism for mechanical property of LY2 aluminum alloy after laser shock processing. Mater. Des..

[107] Li K., Hu Y., Yao Z. (2013). Experimental study of micro dimple fabrication based on laser shock processing. Opt. Laser Technol..

[108] Amar H., Vignal V., Krawiec H., Josse C., Peyre P., da Silva S.N., Dick L.F. (2011). Influence of the microstructure and laser shock processing (LSP) on the corrosion behaviour of the AA2050-T8 aluminium alloy. Corros. Sci..

[109] Zhang Y.K., Ren X.D., Zhou J.Z., Lu J.Z., Zhou L.C. (2009). Investigation of the stress intensity factor changing on the hole crack subject to laser shock processing. Mater. Des..

[110] Vukelić S., Kysar J.W., Yao Y.L. (2009). Grain boundary response of aluminum bicrystal under micro scale laser shock peening. Int. J. Solids Struct..

[111] Gomez-Rosas G., Rubio-Gonzalez C., Ocaña J.L., Molpeceres C., Porro J.A., Morales M., Casillas F.J. (2010). Laser shock processing of 6061-T6 Al alloy with 1064 nm and 532 nm wavelengths. Appl. Surf. Sci..

[112] Yang J.M., Her Y.C., Han N., Clauer A. (2001). Laser shock peening on fatigue behavior of 2024-T3 Al alloy with fastener holes and stopholes. Mater. Sci. Eng. A.

[113] Liu Q., Ding K., Ye L., Rey C., Barter S.A., Sharp P.K., Clark G. (2004). Spallation-like phenomenon induced by laser shock peening surface treatment on 7050 aluminum alloy. Proceedings of the Structural Integrity and Fracture International Conference (SIF'04).

[114] Peyre P., Berthe L., Scherpereel X., Fabbro R. (1998). Laser-shock processing of aluminium-coated 55C1 steel in water-confinement regime, characterization and application to high-cycle fatigue behaviour. J. Mater. Sci..

[115] Lu J., Yang C., Zhang L., Feng A., Jiang Y. (2009). Mechanical properties and microstructure of bionic non-smooth stainless steel surface by laser multiple processing. J. Bionic Eng..

[116] Yilbas B.S., Shuja S.Z., Arif A., Gondal M.A. (2003). Laser-shock processing of steel. J. Mater. Process. Technol..

[117] Luo K.Y., Lu J.Z., Zhang Y.K., Zhou J.Z., Zhang L.F., Dai F.Z., Zhang L., Zhong J.W., Cui C.Y. (2011). Effects of laser shock processing on mechanical properties and micro-structure of ANSI 304 austenitic stainless steel. Mater. Sci. Eng. A.

[118] Ren X.D., Jiang D.W., Zhang Y.K., Zhang T., Guan H.B., Qian X.M. (2010). Effects of laser shock processing on 00Cr12 mechanical properties in the temperature range from 25 to 600 °C. Appl. Surf. Sci..

[119] Sudha C., Parameswaran P., Krishnan R., Dash S., Vijayalakshmi M. (2010). Effect of laser shock processing on the microstructure of 304(L) austenitic stainless steel. Mater. Manuf. Process..

[120] Hu Y., Gong C., Yao Z., Hu J. (2009). Investigation on the non-homogeneity of residual stress field induced by laser shock peening. Surf. Coat. Technol..

[121] Sano Y., Obata M., Yamamoto T. (2006). Residual stress improvement of weldment by laser peening. Weld. Int..

[122] Sano Y., Akita K., Masaki K., Ochi Y., Altenberger I., Scholtes B. (2006). Laser peening without coating as a surface enhancement technology. J. Laser Micro/Nano Eng..

[123] Yakimets I., Richard C., Béranger G., Peyre P. (2004). Laser peening processing effect on mechanical and tribological properties of rolling steel 100Cr6. Wear.

[124] Berthe L., Fabbro R., Peyre P., Braham C., Le J. (2000). Corrosion reactivity of laser-peened steel surfaces. J. Mater. Eng. Perform..

[125] Chu J.P., Rigsbee J.M., Banas G., Elsayed-Ali H.E. (1999). Laser-shock processing effects on surface microstructure and mechanical properties of low carbon steel. Mater. Sci. Eng. A.

[126] Sano Y., Mukai N., Okazaki K., Obata M. (1997). Residual stress improvement in metal surface by underwater laser irradiation. Nucl. Instrum. Methods Phys. Res. B.

[127] Fouinier J., Ballard P., Merien P., Barralis J., Castex L., Fabbro R. (1991). Mechanical effects induced by shock waves generated by high energy laser pulses. J. Phys..

[128] Yoshihiro S., Yuji S., Rie S., Youchul K. (2012). Fatigue life enhancement of fillet and butt welded joints after laser peening. Trans. Join. Weld. Res. Inst. (JWRI).

[129] Chu J., Rigsbee J., Banas G., Lawrence F., Elsayed-Ali H. (1995). Effects of laser-shock processing on the microstructure and surface mechanical properties of Hadfield manganese steel. Metall. Mater. Trans. A.

[130] Gagliardi M.A., Sencer B.H., Hunt A.W., Maloy S.A., Gray G.T. (2011). Relative defect density measurements of laser shock peened 316 L stainless steel using positron annihilation spectroscopy. J. Nondestruct. Eval..

[131] Gerland M., Hallouin M. (1994). Effect of pressure on the microstructure of an austenitic stainless steel shock loaded by very short laser pulses. J. Mater. Sci..

[132] Zhang W., Yao Y.L., Noyan I.C. (2004). Microscale laser shock peening of thin films, part 2: High spatial resolution material characterization. J. Manuf. Sci. Eng..

[133] Wang Y., Fan Y., Vukelic S., Yao Y.L. (2007). Energy-level effects on the deformation mechanism in microscale laser peen forming. J. Manuf. Process..

[134] DeWald A.T., Rankin J.E., Hill M.R., Lee M.J., Chen H.L. (2004). Assessment of tensile residual stress mitigation in alloy 22 welds due to laser peening. J. Eng. Mater. Technol..

[135] Gill A.S., Zhou Z., Lienert U., Almer J., Lahrman D.F., Mannava S.R., Qian D., Vasudevan V.K. (2012). High spatial resolution, high energy synchrotron X-ray diffraction characterization of residual strains and stresses in laser shock peened Inconel 718SPF alloy. J. Appl. Phys..

[136] Fu J., Zhu Y., Zheng C., Liu R., Ji Z. (2014). Effect of laser shock peening on mechanical properties of Zr-based bulk metallic glass. Appl. Surf. Sci..

[137] Lu J.Z., Zhang L., Feng A.X., Jiang Y.F., Cheng G.G. (2009). Effects of laser shock processing on mechanical properties of Fe–Ni alloy. Mater. Des..

[138] Lejia L., Zhong J., Zhenfeng R., Chao Z., Ren L. (1986). Laser shock forming of thin film Fe78Si9B13 metallic. Chin. Sci. Pap..

[139] Grevey D., Maiffredy L., Vannes A. (1992). Laser shock on a TRIP alloy: Mechanical and metallurgical consequences. J. Mater. Sci..

[140] Maawad E., Sano Y., Wagner L., Brokmeier H.G., Genzel C. (2012). Investigation of laser shock peening effects on residual stress state and fatigue performance of titanium alloys. Mater. Sci. Eng. A.

[141] Chen X., Wang J., Fang Y., Madigan B., Xu G., Zhou J. (2014). Investigation of microstructures and residual stresses in laser peened Incoloy 800 H weldments. Opt. Lasers Technol..

[142] Cheng G.J., Cai M., Pirzada D., Guinel M.J.F., Norton M.G. (2008). Plastic Deformation in silicon crystal induced by heat-assisted laser shock peening. J. Manuf. Sci. Eng..

[143] Nalla R., Altenberger I., Noster U., Liu G., Scholtes B., Ritchie R. (2003). On the influence of mechanical surface treatments-deep rolling and laser shock peening on the fatigue behavior of Ti-6Al-4V at ambient and elevated temperatures. Mater. Sci. Eng. A.

[144] Lavender C.A., Hong S.T., Smith M.T., Johnson R.T., Lahrman D. (2008). The effect of laser shock peening on the life and failure mode of a cold pilger die. J. Mater. Process. Technol..

[145] Prevéy P.S. (1986). X-ray diffraction residual stress techniques. Metals Handbook, No. 513.

[146] Prevéy P.S. (1991). Problems with Non-Destructive surface X-Ray diffraction residual stress measurement. Pract. Appl. Residual Stress Technol..

[147] Prevéy P.S. (1986). The use of pearson VII distribution functions in X-Ray diffraction residual stress measurement. Pract. Appl. Residual Stress Technol..

[148] Peyre P., Sollier A., Chaieb I., Berthe L., Bartnicki E., Braham C., Fabbro R. (2003). FEM simulation of residual stresses induced by laser peening. Eur. Phys. J. Appl. Phys..

[149] Luong H., Hill M.R. (2008). The effects of laser peening on high-cycle fatigue in 7085-T7651 aluminum alloy. Mater. Sci. Eng. A.

[150] Gao Y.K. (2011). Improvement of fatigue property in 7050-T7451 aluminum alloy by laser peening and shot peening. Mater. Sci. Eng. A.

[151] Sánchez-Santana U., Rubio-González C., Gomez-Rosas G., Ocaña J.L., Molpeceres C., Porro J., Morales M. (2006). Wear and friction of 6061-T6 aluminum alloy treated by laser shock processing. Wear.

[152] Sano Y., Obata M., Kubo T., Mukai N., Yoda M., Masaki K., Ochi Y. (2006). Retardation of crack initiation and growth in austenitic stainless steels by laser peening without protective coating. Mater. Sci. Eng. A.

[153] Zhao Y. (2012). Effects of Laser Shock Peening on Residual Stress, Texture and Deformation Microstructure of Ti-6Al-4V Alloy. Ph.D. Thesis.

[154] Kalainathan S., Sathyajith S., Swaroop S. (2012). Effect of laser shot peening on precipitation hardened aluminum alloy 6061-T6 using low energy laser. Opt. Lasers Eng..

[155] Ganesh P., Sundar R., Kumar H., Kaul R., Ranganathan K., Hedaoo P., Tiwari P., Kukreja L.M., Oak S.M., Dasari S. (2012). Studies on laser peening of spring steel for automotive applications. Opt. Lasers Eng..

[156] Dorman M., Toparli M.B., Smyth N., Cini A., Fitzpatrick M.E., Irving P.E. (2012). Effect of laser shock peening on residual stress and fatigue life of clad 2024 aluminium sheet containing scribe defects. Mater. Sci. Eng. A.

[157] Ocatia L., Morales M., Molpeceres C., Porro J.A., Abascal J.G., Zupaneic M. (2005). Laser shock processing as a method for surface properties modlficatlon of metallic materials. Proceedings of the 9th International Conference on Shot Peening.

[158] Hu Y., Yao Z., Hu J. (2006). 3-D FEM simulation of laser shock processing. Surf. Coat. Technol..

[159] Clauer J.R., Koucky A.H. (1991). Laser shock processing increases the fatigue life of metal parts. J. Mater. Process..

[160] Hammersley G., Hackel L., Harris F. (2000). Surface prestressing to improve fatigue strength of components by laser shot peening. Opt. Lasers Eng..

[161] Masse G., Barreau J. (1995). Surface modification by laser induced shock waves. Surf. Eng..

[162] Chen H., Kysar J.W., Yao Y.L. (2004). Characterization of plastic deformation induced by microscale laser shock peening. J. Appl. Mech..

[163] Berthe B., Fabbro R., Peyre P. (1999). Wavelength dependent of laser shock-wave generation in the water-confinement regime. J. Appl. Phys..

[164] Hill M., DeWald A., Demma A., Hackel L., Chen H., Dane C., Specht R., Harris F. (2003). Laser peening technology. ASM Int..

[165] Hatamleh O., Smith J., Cohen D., Bradley R. (2009). Surface roughness and friction coefficient in peened friction stir welded 2195 aluminum alloy. Appl. Surf. Sci..

[166] Chandrasekaran K. (1993). On the roughness dependence of wear of steels: A new approach. J. Mater. Sci. Lett..

[167] Rouleau B., Peyre P., Breuils J., Pelletier H., Baudin T., Brisset F. (2011). Characterization at a local scale of a laser-shock peened aluminum alloy surface. Appl. Surf. Sci..

[168] Gomez-Rosas G., Rubio-Gonzalez C., Ocaña J., Molpeceres C., Porro J.A., Chi-Moreno W., Morales M. (2005). High level compressive residual stresses produced in aluminum alloys by laser shock processing. Appl. Surf. Sci..

[169] Chahardehi A., Brennan F.P., Steuwer A. (2010). The effect of residual stresses arising from laser shock peening on fatigue crack growth. Eng. Fract. Mech..

[170] Heckenberger U.C., Hombergsmeier E., Holzinger V., von Bestenbostel W. (2011). Laser shock peening to improve the fatigue resistance of AA7050 components. Int. J. Struct. Integr..

[171] Ganesh P., Sundar R., Kumar H., Kaul R., Ranganathan K., Hedaoo P., Raghavendra G., Anand Kumar S., Tiwari P., Nagpure D.C. (2014). Studies on fatigue life enhancement of pre-fatigued spring steel specimens using laser shock peening. Mater. Des..

[172] Banas G., Lawrence F.V. (1990). Shot peening *vs.* laser shock peening. ICSP4.

[173] Shepard M.J., Prevéy P.S., Jayaraman N. (2003). Effects of surface treatment on fretting fatigue performance of Ti-6Al-4V. Proceedings of the 8th National Turbine Engine High Cycle Fatigue (HCF) Conference.

[174] Zhang X., Liu D. (2009). Effect of TiN/Ti multilayer on fretting fatigue resistance of Ti-811 alloy at elevated temperature. Trans. Nonferrous Met. Soc. China.

[175] King A., Steuwer A., Woodward C., Withers P.J. (2006). Effects of fatigue and fretting on residual stresses introduced by laser shock peening. Mater. Sci. Eng. A.

[176] Scherpereel N., Peyre P., Fabbro R., Lederer G., Celati N. (1997). Modifications of mechanical and electrochemical properties of stainless steel surfaces by laser shock processing. Proc. SPIE.

[177] Yoda M., Newton B. (2008). Underwater Laser Peening. Proceedings of the Welding and Repair Technology for Power Plants Eighth International EPRI Conference.

[178] Hatamleh O., Singh P.M., Garmestani H. (2008). Stress corrosion cracking behavior of peened friction stir welded 2195 aluminum alloy joints. J. Mater. Eng. Perform..

[179] Hackel L., Rankin J., Dane C.B., Harris F. (2012). Mitigation of Stress corrosion cracking and cavitation erosion in ship structures and systems by laser peening. Curtiss Wright Surface Technologies.

[180] Prevéy P., Hornbach D., Mason P. (1998). Thermal residual stress relaxation and distortion in surface enhanced gas turbine engine components. Proceedings of the 17th Heat Treating Society Conference and Exposition and the 1st International Induction Heat Treating Symposium.

[181] Nikitin I., Scholtes B., Maier H., Altenberger I. (2004). High temperature fatigue behavior and residual stress stability of laser-shock peened and deep rolled austenitic steel AISI 304. Scr. Mater..

[182] Ye C., Liao Y., Suslov S., Lin D., Cheng G.J. (2014). Ultrahigh dense and gradient nano-precipitates generated by warm laser shock peening for combination of high strength and ductility. Mater. Sci. Eng. A.

[183] Lin D., Ye C., Liao Y., Suslov S., Liu R., Cheng G.J. (2013). Mechanism of fatigue performance enhancement in a laser sintered superhard nanoparticles reinforced nanocomposite followed by laser shock peening. J. Appl. Phys..

[184] Clauer A. (2013). Laser shock processing: Past, present and future. Proceedings of the 4th International Conference on Laser Peening.

[185] Kamkarrad H., Narayanswamy S., Tao X.S. (2014). Feasibility study of high-repetition rate laser shock peening of biodegradable magnesium alloys. Int. Adv. Manuf. Technol..

[186] Braisted W., Brockman R. (1999). Finite element simulation of laser shock peening. Int. J. Fatigue.

[187] Tsuji N., Tanaka S., Takasugi T. (2009). Effects of combined plasma-carburizing and shot-peening on fatigue and wear properties of Ti-6Al-4V alloy. Surf. Coat. Technol..

[188] Achintha M., Nowell D., Shapiro K., Withers P.J. (2013). Eigenstrain modelling of residual stress generated by arrays of laser shock peening shots and determination of the complete stress field using limited strain measurements. Surf. Coat. Technol..

[189] Voothaluru R., Liu C.R., Cheng G.J. (2012). Finite element analysis of the variation in residual stress Distribution in laser shock peening of steels. J. Manuf. Sci. Eng..

[190] Zhang W., Yao Y.L., Noyan I.C. (2004). Microscale laser shock peening of thin films, part 1: Experiment, modeling and simulation. J. Manuf. Sci. Eng..

[191] Ding K., Ye L. (2006). Simulation of multiple laser shock peening of a 35CD4 steel alloy. J. Mater. Process. Technol..

[192] Morales M., Correa C., Porro J.A., Molpeceres C., Ocaña J.L. (2011). Thermomechanical modelling of stress fields in metallic targets subject to laser shock processing. Int. J. Struct. Integr..

[193] Korsunsky A.M. (2006). Residual elastic strain due to laser shock peening: Modelling by eigenstrain distribution. J. Strain Anal. Eng. Des..

[194] Wang Y., Kysar J.W., Yao Y.L. (2008). Analytical solution of anisotropic plastic deformation induced by micro-scale laser shock peening. Mech. Mat..

[195] Wu B., Shin Y.C. (2007). A one-dimensional hydrodynamic model for pressures induced near the coating-water interface during laser shock peening. J. Appl. Phys..

[196] Vukelić S., Wang Y., Kysar J.W., Yao Y.L. (2009). Dynamic material response of aluminum single crystal under microscale laser shock peening. J. Manuf Sci. Eng..

[197] Ren N.F., Yang H.M., Yuan S.Q., Wang Y., Tang S.X., Zheng L.M., Ren X.D., Dai F.Z. (2014). High temperature mechanical properties and surface fatigue behavior improving of steel alloy via laser shock peening. Mater. Des..

[198] Ye C., Suslov S., Lin D., Liao Y., Cheng G.J. (2014). Cryogenic ultrahigh strain rate deformation induced hybrid nanotwinned microstructure for high strength and high ductility. J. Appl. Phys..

[199] Yu C., Gao H., Yu H., Jiang H., Cheng G.J. (2009). Laser dynamic forming of functional materials laminated composites on patterned three-dimensional surfaces with applications on flexible microelectromechanical systems. Appl. Phys. Lett..

[200] Bossi R., Housen K., Walters C. (2005). Laser bond inspection device for composites. NTIAC (Nondestruct. Test. Inform. Anal. Center) Newsl..

[201] Budinski K.G., Budinski M.K. (2010). Engineering Materials-Properties and Selection.

[202] Robinson J.I., Reed R.C. (1995). Water droplet erosion of laser surface treated Ti-6A1-4V. Wear.

[203] Rockstroh T.J., Bailey M.S., Ash C.A., Ulanski W. (2008). Laser Shock Processing of Aircraft Engine Components.

[204] Fan Y., Wang Y., Vukelic S., Yao Y.L. (2007). Numerical investigation of opposing dual sided microscale laser shock peening. J. Manuf. Sci. Eng..

[205] Lin B., Lupton C., Spanrad S., Schofield J., Tong J. (2014). Fatigue crack growth in laser shock peened Ti-6Al-4V aerofoil specimens due to foreign object damage. Int. J. Fatigue.

[206] Shepard M.J., Smith P.R., Amer M.S. (2001). Introduction of compressive residual stresses in Ti-6Al-4V simulated airfoils via laser shock processing. J. Mater. Eng. Perform..

[207] Spanrad S., Tong J. (2011). Characterisation of foreign object damage (FOD) and early fatigue crack growth in laser shock peened Ti-6Al-4V aerofoil specimens. Mater. Sci. Eng. A.

[208] Amarchinta H.K., Grandhi R.V., Langer K., Stargel D.S. (2009). Material model validation for laser shock peening process simulation. Modell. Simul. Mater. Sci. Eng..

